# Use of Half-Generation PAMAM Dendrimers (G0.5–G3.5) with Carboxylate End-Groups to Improve the DACHPtCl_2_ and 5-FU Efficacy as Anticancer Drugs †

**DOI:** 10.3390/molecules26102924

**Published:** 2021-05-14

**Authors:** Cláudia Camacho, Helena Tomás, João Rodrigues

**Affiliations:** 1 CQM-Centro de Química da Madeira, MMRG, Universidade da Madeira, Campus da Penteada, 9000-390 Funchal, Portugal; ccamacho@staff.uma.pt (C.C.); lenat@staff.uma.pt (H.T.); 2 School of Materials Science and Engineering, Center for Nano Energy Materials, Northwestern Polytechnical University, Xi’an 710072, China

**Keywords:** dendrimers, PAMAM, anticancer drugs, metallodrugs, oxaliplatin, 5-FU

## Abstract

The DACHPtCl_2_ compound (*trans*-(R,R)-1,2-diaminocyclohexanedichloroplatinum(II)) is a potent anticancer drug with a broad spectrum of activity and is less toxic than oxaliplatin (*trans*-l-diaminocyclohexane oxalate platinum II), with which it shares the active metal fragment DACHPt. Nevertheless, due to poor water solubility, its use as a chemotherapeutic drug is limited. Here, DACHPtCl_2_ was conjugated, in a bidentate form, with half-generation PAMAM dendrimers (G0.5–G3.5) with carboxylate end-groups, and the resulting conjugates were evaluated against various types of cancer cell lines. In this way, we aimed at increasing the solubility and availability at the target site of DACHPt while potentially reducing the adverse side effects. DNA binding assays showed a hyperchromic effect compatible with DNA helix’s disruption upon the interaction of the metallodendrimers and/or the released active metallic fragments with DNA. Furthermore, the prepared DACHPt metallodendrimers presented cytotoxicity in a wide set of cancer cell lines used (the relative potency regarding oxaliplatin was in general high) and were not hemotoxic. Importantly, their selectivity for A2780 and CACO-2 cancer cells with respect to non-cancer cells was particularly high. Subsequently, the anticancer drug 5-FU was loaded in a selected metallodendrimer (the G2.5COO(DACHPt)_16_) to investigate a possible synergistic effect between the two drugs carried by the same dendrimer scaffold and tested for cytotoxicity in A2780cisR and CACO-2 cancer cell lines. This combination resulted in IC_50_ values much lower than the IC_50_ for 5-FU but higher than those found for the metallodendrimers without 5-FU. It seems, thus, that the metallic fragment-induced cytotoxicity dominates over the cytotoxicity of 5-FU in the set of considered cell lines.

## 1. Introduction

**Figure 1:**
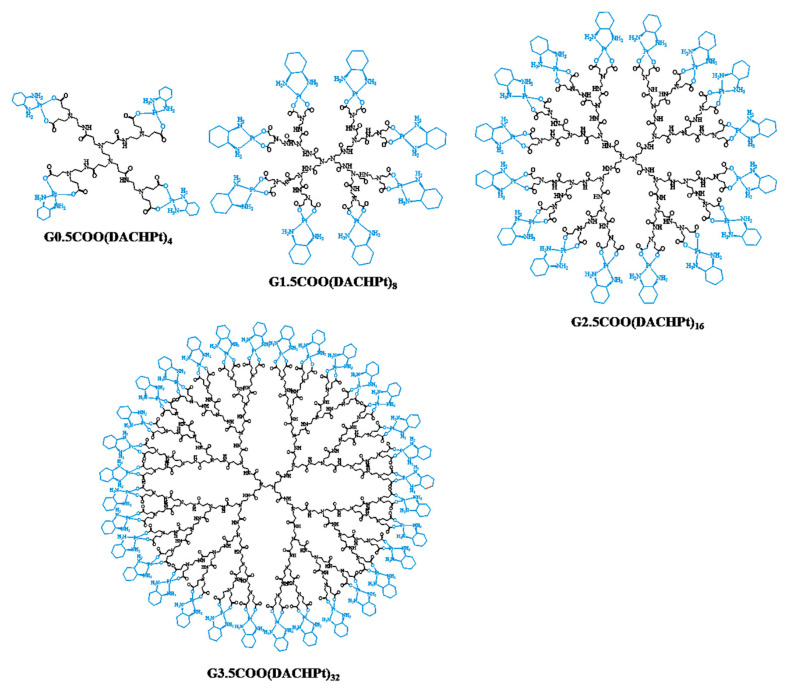
Prepared PAMAM dendrimers with carboxylate end-groups (half-generations G0.5 to G3.5) functionalized with DACHPt moiety.

*Trans*-l-diaminocyclohexane oxalate platinum(II) (oxaliplatin) is a chemotherapeutic drug used in the frontline to treat colorectal cancer (among others) due to its exceptional activity [1,2]. This third-generation platinum anticancer drug was developed to overcome cellular resistance to cisplatin [3,4]. Oxaliplatin is a square planar platinum(II) complex that has a chiral bidentate ligand, the *trans*-(R,R)-1,2-diaminocyclohexane (DACH), and an oxalate leaving group. The DACHPt unit of oxaliplatin plays an important role in cytotoxicity once the DACH chiral and bulky ligand induces a conformational distortion in DNA different from that caused by cisplatin, forming adducts that are more hydrophobic and distinctively interfere with DNA replication and transcription [5,6,7,8,9,10,11,12]. Indeed, the main mechanism of action is the formation of DNA-adducts, which implies oxaliplatin hydrolysis and the formation of reactive species that will interact with DNA, being the 1,2-intrastrand crosslinks the most prevalent adducts [7,13,14,15]. Despite its efficacy in treating colorectal cancer, the associated side effects of oxaliplatin, such as neurotoxicity, can be severe and limit the dose applied to the patient, which may compromise the therapy efficacy [3,16,17,18]. Oxalate, the leaving group in oxaliplatin, is a recognized calcium chelator related to its neurotoxicity effects [19]. Importantly, oxaliplatin is usually used in a combination regimen with other chemotherapeutic drugs to treat stage III of colorectal cancer and their recurrences, such as the chemoprotectant reduced form of folic acid, the 5-formyl tetrahydrofolic acid (Leucovorin), and the 5-fluoro-1*H*,3*H*-pyrimidine-2,4-dione, known as Fluorouracil (5-FU) (usually known as FOLFOX: FOLinic acid-Fluororuracil-OXaliplatin chemotherapy regimen for colorectal cancer treatment). The combination with 5-FU is often used and showed a synergistic effect in the treatment, even in tumors resistant to oxaliplatin [2,6,16]. In this context, based on our team expertise in the domain of dendrimers chemistry for biomedical applications [20,21,22,23,24], we decided to explore the possibility of using anionic PAMAM dendrimers as nanocarriers for the DACHPt active fragment. This strategy eliminates the need to use oxalate as a drug component and, in addition, allows us to deliver the 5-FU anticancer drug simultaneously. For this purpose, several generations of PAMAM dendrimers with carboxylate end-groups (half-generations G0.5 to G3.5) were functionalized with DACHPt moiety (Figure 1), characterized by suitable physicochemical techniques (NMR, MS, FTIR, UV–vis and fluorescence spectroscopy), and their biological behavior was evaluated in vitro. Then, the metallodendrimer G2.5COO(DACHPt)_16_ was selected to carry 5-FU molecules, and the effect of the combination of the two drugs was also studied. As far as we know, this is the first study that integrates the active fragment of oxaliplatin and the 5-FU drug together in a dendrimer scaffold as a new approach for the simultaneous delivery of these two drugs aiming at reducing the side effects, such as neurotoxicity and neuropathy.

## 2. Results and Discussion

### 2.1. Synthesis and Characterization of DACHPt Metallodendrimers

For the preparation of DACHPt metallodendrimers, the *trans*-(R,R)-1,2-diaminocyclohexanedichloroplatinum(II), DACHPtCl_2_, was first synthesized with 59% yield and characterized by different techniques, such as NMR, FTIR, UV–visible, and fluorescence spectroscopy. The characteristic signals of DACHPtCl_2_ corresponding to the cyclohexyl and amine protons [25,26,27] can be seen in the ^1^H-spectrum (Figure S1). The FTIR spectrum (Figure S2) also presents the characteristic bands of the DACHPtCl_2_ compound, namely the N–H stretch of amine groups at 3276 cm^−1^ and 3186 cm^−1^, and the N–H bend at 1566 cm^−1^. Bands corresponding to the C–H stretch were also observed at 2865 cm^−1^ and 2935 cm^−1^ [28]. In the UV–vis spectra (Figure S3a), a shoulder with a maximum in the UV region (212 nm) appears, due, probably, to a mixture with the characteristic charge transfer transitions of the halide platinum (II) complexes with shifted d–d transitions [29]. The fluorescence emission spectrum (Figure S3b) shows a band at around 429 nm due to the cyclohexyl group of DACHPtCl_2_. Afterward, to attach the DACHPt fragment to the PAMAM dendrimers, the aquation of DACHPtCl_2_ was necessary to form reversible coordinated bonds with the carboxylate end groups of PAMAM dendrimer half-generations. The aquation of DACHPtCl_2_ is also important to guarantee the removal of the two chlorines and assure the conjugation of the DACHPt moiety to the anionic PAMAM dendrimer in the bidentate form. Indeed, the conjugation of the DACHPt fragment to the dendrimer involving two bonds (bidentate form) should delay drug release before the target is achieved, thus decreasing the side effects. As so, bis-aquated DACHPtCl_2_ was synthesized through a reaction of DACHPtCl_2_ with silver nitrate, an abstractor of chloride ligands, with 86% yield. The product was characterized by ^1^H and ^13^C-NMR spectroscopy (Figures S4 and S5).

**Figure 2:**
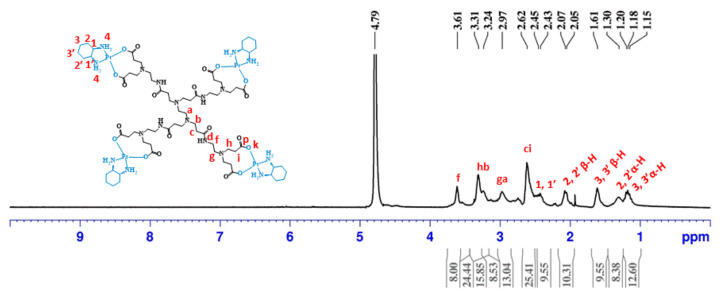
^1^H-NMR spectrum of G0.5COO(DACH)Pt_4_ performed in D_2_O.

After, four half-generations of anionic PAMAM dendrimers (G0.5–G3.5) were successfully coordinated to the bis-aquated DACHPtCl_2_ in a bidentate form. The prepared metallodendrimers were obtained with a good yield (G0.5COO(DACHPt)_4_: 92%; G1.5COO(DACHPt)_8_: 77%; G2.5COO(DACHPt)_16_: 78%; G3.5COO(DACHPt)_32_: 60%). Again, the products were fully characterized by different techniques that included NMR (^1^H, ^13^C, and ^1^⁹⁵Pt-NMR), FTIR, UV–vis, and fluorescence spectroscopy, zeta-potential, and mass spectrometry. In the ^1^H-NMR experiments, the signal of deuterated water (D_2_O) was used as an internal reference, whereas potassium tetrachloroplatinate(II) was used as an external reference in the ^1^⁹⁵Pt-NMR experiments. Figure 2 shows the expected ^1^H-NMR spectrum of G0.5COO(DACHPt)_4_ with the characteristic signals of the anionic PAMAM dendrimer structure between 2.62 and 3.61 ppm and the signals of the cyclohexyl group of DACHPt fragment between 2.43 and 1.18 ppm.

**Figure 3:**
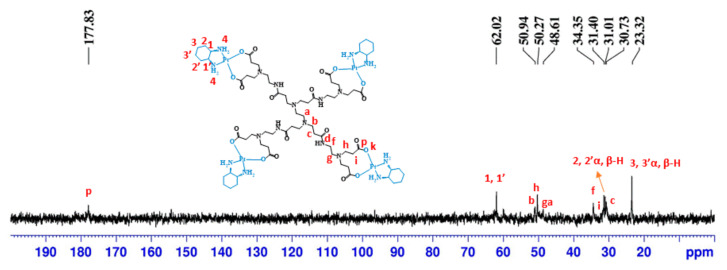
^13^C-NMR spectrum of G0.5COO(DACH)Pt_4_ performed in D_2_O.

The ^13^C-NMR spectrum (Figure 3) also presents the characteristic signals of the anionic PAMAM dendrimer’s structure and those corresponding to the cyclohexyl of the DACHPt moiety. The signals at 30.73, 31.40, 34.35, 48.61, 50.27, and 50.94 ppm are from the anionic PAMAM dendrimer scaffold. The signals at 23.32, 31.01, and 62.02 ppm are from the cyclohexyl group of DACHPt. Compared to pristine half-generation anionic PAMAM dendrimers, a downfield shift was observed in the carboxylate group’s signal (from 174.69 to 177.83 ppm), indicative of metal complex coordination [30]. Moreover, the coordination of DACHPt to the anionic PAMAM dendrimers was evaluated by TOF-MS (ESI positive mode or MALDI, Table S1 and Figures S6–S9). Despite the characterization difficulties experienced since DACHPt metallodendrimers are, in general, hygroscopic, several fragments from the parent metallodendrimers were identified by TOF-MS, confirming the functionalization with DACHPt. (See Section 3.2).

Furthermore, the signal at −2314 ppm in the ^1^⁹⁵Pt-NMR spectrum (Figure S10), although having low intensity and high noise due to the sensitivity of the NMR probe for the ^1^⁹⁵Pt-nucleus, also suggests that the DACHPt moiety was conjugated to the PAMAM dendrimer in a bidentate form [31,32]. Similar results were observed for the other dendrimer generations, as shown in Figures S11–S19. The prepared compounds were also characterized by FTIR to confirm the formation of the DACHPt metallodendrimers with the DACHPt moiety conjugated in the bidentate form. When compared with the respective pristine anionic PAMAM dendrimer (Figure S20), the DACHPt metallodendrimers (G0.5COO(DACHPt)_4,_ G1.5COO(DACHPt)_8,_ G2.5COO(DACHPt)_16,_ and G3.5COO(DACH)Pt_32_) show the characteristic carbonyl stretching band in the range 1618 cm^−1^ to 1640 cm^−1^ (Figure S21) with the N–H stretch of the NH_2_ groups of the DACHPt fragment appearing in the range 3388 cm^−1^ to 3470 cm^−1^. In the G0.5COO(DACHPt)_4_ spectrum, a shift to a lower wavenumber on the carbonyl group band (C=O stretch), from 1639 cm^−1^ to 1618 cm^−1^, is observable, being indicative of the bidentate complexation of the DACHPt fragment via the dendrimer carboxyl terminal groups [33]. In the other DACHPt metallodendrimers, this shift is difficult to observe, but the conjugation could be confirmed due to the N–H stretch of the NH_2_ groups of the DACHPt fragment.

**Figure 4:**
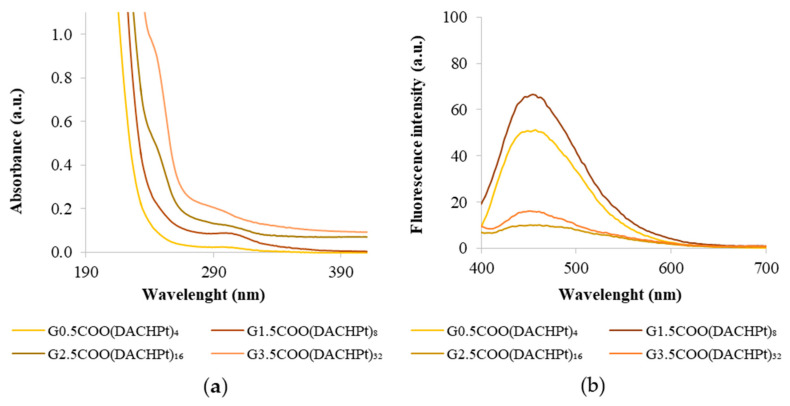
(**a**) UV–vis spectra of DACHPt metallodendrimers at a concentration of 40 µM and (**b**) emission (λ_ex_ = 380 nm) of DACHPt metallodendrimers at a concentration of 500 µM in UPW.

As mentioned earlier, the DACHPtCl_2_ complex has a maximum absorption at 212 nm. After the conjugation of DACHPt to the dendrimer, a shoulder around this wavelength is observed in the UV-vis spectra, more evident for the higher generation dendrimers. The maximum absorbance wavelength suffers a deviation for higher wavelengths as the dendrimer generation increases, which is additional evidence of the success of the coordination process (Figure 4a). Moreover, the characteristic absorption band of the half-generation anionic PAMAM dendrimers, which appears around 280–300 nm and that is attributed to the interior tertiary amines (Figure S22), remains visible, without any significant shift, indicating that DACHPt has been conjugated only at the surface of the PAMAM dendrimers. As the generation of the dendrimers increases, absorbance values become higher due to the increase in the number of tertiary amines.

It is known that PAMAM dendrimers possess intrinsic fluorescence properties [20,34]. Indeed, the emission spectra presented by the pristine anionic PAMAM dendrimers show maximum wavelengths of fluorescence in the range of 440–455 nm (Figure S23). Interestingly, after the dendrimers’ coordination of the DACHPt fragment, the fluorescence intensity decreased considerably in this wavelength range (Figure 4b). This effect occurs notwithstanding the DACHPtCl_2_ compound and also shows fluorescence in an aqueous solution with a maximum emission band at 433 nm (Figure S3). This result may be due to a decrease in the intrinsic fluorescence of dendrimers upon coordination. The bidentate coordination that occurs may affect the distance among branches within the dendrimer scaffold interfering with the overall rigidity in the dendrimer scaffold or/and to quenching effects related to the proximity of the DACHPt peripheral groups.

Furthermore, after coordination of the half-generation anionic PAMAM dendrimers to the DACHPt moiety, the zeta-potential values become less negative for all the synthesized metallodendrimers (Table 1). This observation was expected since the dendrimers are anionic, and the DACHPt cation fragment has an associated double positive charge. Nevertheless, it is important to stress that, even if zeta-potential is not an absolute indication regarding the stability of nanoparticles, the observed reduction in its absolute value after functionalization of the dendrimers with the DACHPt unit may trigger dendrimers’ aggregation in an aqueous solution.

### 2.2. DNA Binding Assays

**Figure 5:**
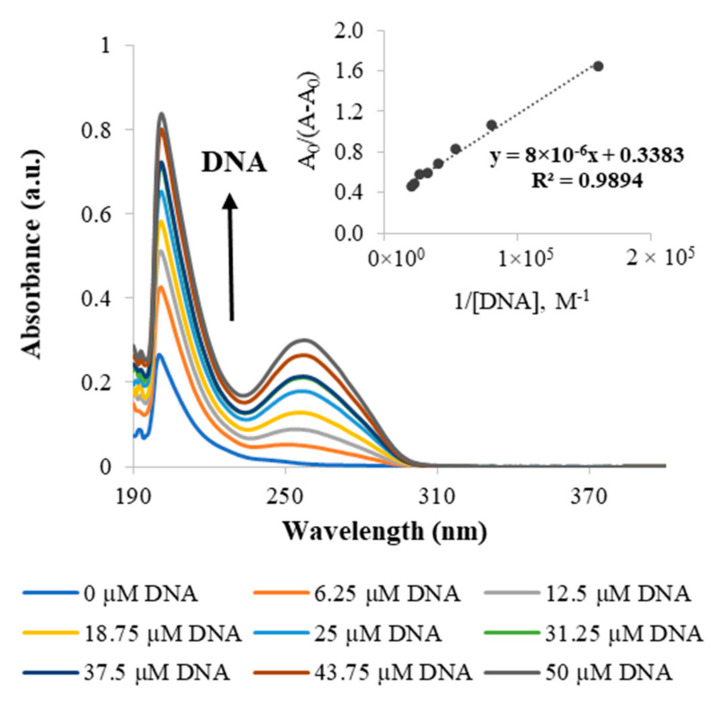
Representative UV–visible spectrum of the G2.5COO(DACHPt)_16_ metallodendrimer with increasing concentrations of CT-DNA (0, 6.25, 12.5, 18.75, 25, 31.25, 37.5, 43.75 and 50 µM) in 5 mM Tris-HCl/50 mM NaCl at pH 7.4. The inset corresponds to the plot of A_0_/(A−A_0_) versus 1/[DNA], which is used to determine the binding constant. The arrow indicates the direction of increasing the concentration of DNA.

Since DNA is the pharmacological target of platinum-based drugs, UV–vis spectroscopy was used to study the in vitro interaction of the metallodendrimers with calf thymus DNA (CT-DNA). The DNA binding studies were performed for the G2.5COOPt(DACHPt)_16_ metallodendrimer, as well as for DACHPtCl_2_ and oxaliplatin for comparison purposes. This metallodendrimer was selected as a model for this family of metallodendrimer since, due to its size, it seemed more appropriate for the loading of 5-FU, which was later performed (see Section 2.3). Figure 5 shows the effect on the absorption spectra of solutions containing a varying CT-DNA concentration and a constant concentration of metallodendrimer. The CT-DNA spectrum shows a broadband in the UV region with a maximum absorption wavelength at 260 nm. This absorption is due to the chromophoric groups of adenine, guanine, cytosine, and thymine. It has been described in the literature that whereas binding to DNA through intercalation results in hypochromism, binding by electrostatic interactions gives rise to a hyperchromic effect (an increase in DNA absorption). Hyperchromism may also arise from other causes that culminate in a disruption of the hydrogen bonds that keep the DNA double helix in place and limit the resonance of the aromatic rings (limiting absorption as well). Clearly, the spectra of the G2.5COO(DACHPt)_16_ metallodendrimer in the presence of increasing CT-DNA concentrations present a hyperchromic effect. The same was also observed for the DACHPtCl_2_ moiety and oxaliplatin (Figures S24 and S25, respectively). Since it is known that the active fragment of oxaliplatin forms covalent adducts with DNA, the observed hyperchromism in DACHPtCl_2_ and oxaliplatin should be due to a distortion in DNA conformation caused by adduct formation that exposes the DNA bases and results in higher absorbance values. These results are consistent with other studies regarding the interaction of oxaliplatin with linear DNA [35]. In the case of the G2.5COO(DACHPt)_16_ metallodendrimer, one should not expect a full release of the coordinated metallic fragments from the dendrimer scaffold during the 5 min incubation period used in the assay, even in the presence of chloride ions. If that happened, the dendrimeric scaffold was always present in the solution. For the metallodendrimer, other types of interactions may occur, such as those of a solely electrostatic nature that may further contribute to the DNA helix’s disruption.

The DNA binding constant (K_b_) of the compounds with CT-DNA was determined by UV-vis through the Benesi–Hildebrand equation, namely from the ratio of the y-intercept to the slope in the plots A_0_/A−A_0_ vs. 1/[DNA] [36]. The found K_b_ values were similar for DACHPtCl_2_ and oxaliplatin (≃3 × 10^3^ M^−1^), but a much higher value was determined for the metallodendrimer (Table 2). The coordination of the PAMAM dendrimer to the DACHPt increased the K_b_ for (3.6 ± 0.9) × 10^4^ M^−1^, reflecting a strong interaction of the metallodendrimers with the CT-DNA in the performed in vitro experiments. As mentioned above, additional electrostatic interactions may contribute to strengthen these interactions. However, one should have in mind that the type and extent of electrostatic interactions between the metallodendrimer and the CT-DNA established in vitro cannot directly correlate with what will happen in vivo. In vivo, the release of the metallic fragments is expected to occur near the target site (in the tumor environment or even inside cancer cells). In addition, the Gibbs free energy of the binding process was also estimated, revealing that it was a spontaneous process as negative values were obtained (Table 2).

### 2.3. Biological Studies

#### 2.3.1. In Vitro Cytotoxicity Assays

The cytotoxicity of the metallodendrimers G0.5COO(DACHPt)_4_, G1.5COO(DACHPt)_8_, G2.5COO(DACHPt)_16_, G3.5COO(DACHPt)_32,_ and the free drugs DACHPtCl_2_ and oxaliplatin were studied in vitro using four cancer cell lines (A2780, A2780CisR, MCF-7, and CACO-2 cells), and one non-cancer cell line (BJ cells). The cytotoxicity was evaluated through a metabolic activity assay (MTT assay) after 72 h, and the results are presented as the half-maximal inhibitory concentration (IC_50_) in Table 3. All the studied metallodendrimers were toxic against the tested cancer cell lines. Furthermore, the metallodendrimers were more cytotoxic than oxaliplatin and DACHPtCl_2_ for all the cancer cell lines considered, as reflected by the obtained IC_50_ values.

This increase in cytotoxicity can be more easily understood by analyzing the cytotoxicity relative potential (RP) of the metallodendrimers regarding oxaliplatin. This RP parameter was determined by dividing the oxaliplatin IC_50_ by the metallodendrimers IC_50_ (Table 4). As can be seen, RP values were consistently higher than 1, thus revealing a higher anticancer activity of the metallodendrimers concerning oxaliplatin. The A2780 and the MCF-7 cells were the most sensitive to the metallodendrimers. This higher cytotoxicity of the metallodendrimers must be due to the higher number of metallic fragments transported by the dendrimer scaffold compared with the single metallic fragment present in the oxaliplatin molecule. However, apparently, an effect of dendrimer generation was not observed in the cytotoxic behavior of the metallodendrimers.

Selectivity for cancer cells is another important characteristic of a chemotherapeutic agent, and, as such, the cytotoxicity of the metallodendrimers was also studied using non-cancer BJ cells. The selectivity indexes (SIs) of the metallodendrimers are presented in Table 5 for the different cancer cell lines used in the experiments (the ratio between the IC_50_ for BJ cells and the IC_50_ for each cancer cell line). It is considered that if the SI is greater than 2, then the more selective the compound is towards cancer cells. SI values less than or equal to 2 mean that the compound has only general toxicity [37,38,39]. The results show that the metallodendrimers are especially selective regarding A2780 cancer cells. Importantly, the metallodendrimers also show selectivity for CACO-2 cancer cells.

The development of new platinum-based anticancer drugs also aimed to overcome the resistance to cisplatin (the first of this class of drugs that was introduced in the clinic) developed by some cell types. For this reason, we carried out the experiments with the A2780 cell line and also with its corresponding variant resistant to cisplatin, the A2780CisR cell line. The resistance factor (RF), which is the ratio between the IC_50_ value for A2780cisR cells and the IC_50_ value for A2780 cells, was then used as a parameter to evaluate the capacity to surpass cisplatin resistance (Table 6). Thus, the lower the RF value, the better the compound to overcome the resistance. However, when compared to free drugs, our metallodendrimers showed high RF values, which means that they have no advantages in this regard [40].

To conclude, considering previous work on the field, such as the seminal work of Ruth Duncan et al. [41], Hitesh Kulhari [42], and particularly of Gordon Kirkpatrick [40] on the preparation of PAMAM dendrimers functionalized with cisplatin, despite the precaution needed comparing in vitro studies, our metallodendrimers with DACHPt unit, tested in A2780 and A2780CisR cells, presented IC_50_ results that are, in general, 18–49 times lower than the reported for cis[Pt(NH_3_)_2_]-G3.5-G6.5 [40]. They apparently demonstrate a higher efficacy, confirming the better behavior of the lower metallodendrimers generations and, at this level, the superior performance of DACHPt fragment over the conjugation of PAMAM with cisplatin.

#### 2.3.2. Hemotoxicity Assays

**Figure 6:**
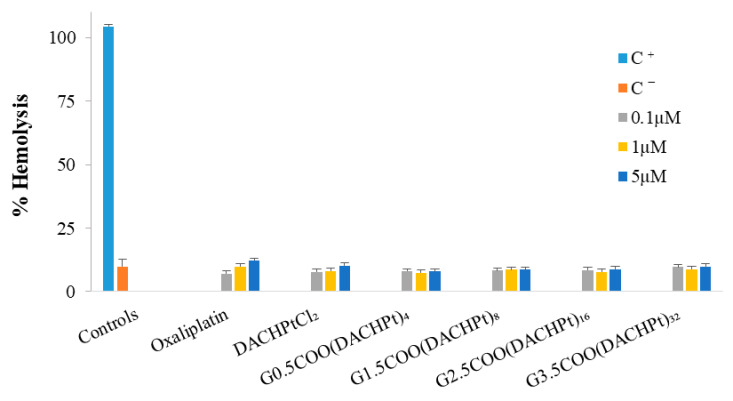
Hemotoxicity of the free DACHPtCl_2_, oxaliplatin, and the prepared DACHPt metallodendrimers. Blood was treated for 3 h with different concentrations (0.1 µM, 1 µM, and 5 µM) of the metallodendrimers and free drugs. The positive and negative control are represented by C^+^ and C^−^, respectively. The results are expressed as mean ± SD of at least three independent experiments performed in triplicate.

The hemolysis assay was used to evaluate the interaction of the free drugs and DACHPt metallodendrimers with red blood cells. The obtained results (Figure 6) show very low hemotoxicity levels for all situations studied if we compare the values of released hemoglobin with those of the negative control. In the case of oxaliplatin, hemotoxicity slightly increases with concentration with a release of hemoglobin around 12% at the maximum concentration used (5 µM), which is in line with the reported oxaliplatin hematological toxicity [7]. At this concentration, both the DACHPtCl_2_ and the metallodendrimers also present a low hemolysis percentage (7–10%). In fact, at least in the concentration range studied, an increase in dendrimers generation and, consequently, an increase in the number of metallic centers does not induce a significant variation in hemotoxicity values.

However, these hemotoxicity studies were carried out in vitro and, of course, do not reflect the totality of events that may occur in vivo, such as selective dendrimers aggregation on atheromatous carotid tissues [43], platelets aggregation [44], and toxic effects in hippocampal neurons that may lead to a significant reduction in viability [45] as reported by others regarding dendrimer-based nanomaterials.

### 2.4. Drug Loading

#### 2.4.1. Loading of 5-FU

As stated in the introduction, 5-FU is part of the chemotherapy regimen used to treat colorectal cancer, and, as such, it was chosen as a second drug to be transported by the anionic PAMAM dendrimers. The metallodendrimer G2.5COO(DACHPt)_16_ was selected to load 5-FU due to its size, which seemed adequate for drug encapsulation. Drug loading results were compared with those of the pristine dendrimer G2.5(COONa)_32_. The G2.5COO(DACHPt)_16_ had a loading efficiency of 75%, which was slightly lower compared to the G2.5(COONa)_32_ that had a loading efficiency of 86% (Table 7). In addition, the loading capacity in the G2.5COO(DACHPt)_16_/5-FU system was also lower (14%), which corresponds to a lower number of encapsulated 5-FU molecules (11). The loading capacity of the pristine G2.5(COONa)_32_ dendrimer was more than two times that shown by the metallodendrimer (ca. 32%).

**Figure 7:**
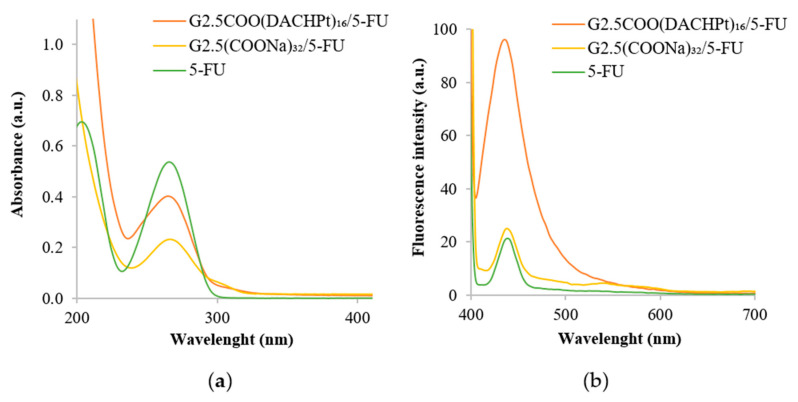
(**a**) UV–vis spectra of G2.5COO(DACHPt)_16_/5-FU, G2.5(COONa)_32_/5-FU, and 5-FU (**b**) emission spectra (λ_ex_ = 380 nm) of G2.5COO(DACHPt)_16_/5-FU, G2.5(COONa)_32_/5-FU and 5-FU. In UPW, spectra were recorded at the same 5-FU concentration ((5-FU) = 10 µg).

Moreover, the UV–vis spectrum of the G2.5COO(DACHPt)_16_/5-FU system was recorded. The 5-FU encapsulation in the metallodendrimer was confirmed (Figure 7a) by a decrease observed in absorbance intensity at 266 nm, which is the maximum absorption wavelength of 5-FU [46]. The decrease in the absorbance was also observed in the case of G2.5(COONa)_32_. Furthermore, fluorescence emission spectra were also performed. As can be seen in Figure 7b, 5-FU and G2.5(COONa)_32_/5-FU present similar fluorescence intensity, whereas it is much higher for the G2.5COO(DACHPt)_16_/5-FU system (in this case, fluorescence intensity is five times greater than in the G2.5(COONa)_32_/5-FU system).

As can be observed from Table 8, the zeta-potential increases from −10.8 mV in the metallodendrimer G2.5COO(DACHPt)_16_ to 0.8 mV in the G2.5COO(DACHPt)_16_/5-FU system, corroborating the loading of 5-FU in the G2.5COO(DACHPt)_16_. A similar trend was observed in the zeta-potential when the G2.5(COONa)_32_ dendrimers were loaded with 5-FU.

The FTIR spectrum of G2.5COO(DACHPt)_16_/5-FU is shown in Figure S26. A shift in the characteristic absorption bands of the G2.5COO(DACHPt)_16_ metallodendrimer is observed after encapsulation of 5-FU. The amide I band C=O stretching at 1640 cm^−1^ moves to 1644 cm^−1^, and the amide II band, due to the N–H bending from 1560 cm^−1^ to 1586 cm^−1^. This shift is due to hydrogen bonding between the 5-FU and the metallodendrimer [47]. Furthermore, shifts in the signals of 5-FU carbonyl group C=O (from 1658 cm^−1^ to 1644 cm^−1^) and of the C–F stretching band (from 1244 cm^−1^ to 1262 cm^−1^) are visible [48]. Likewise, a shift of amide I band, from 1638 cm^−1^ to 1644 cm^−1,^ and amide II band from 1564 cm^−1^ to 1586 cm^−1^ is observed for the G2.5(COONa)_32_/5-FU after the drug encapsulation. (Figure S27). In addition to the characterizations carried out, the complexes G2.5COO(DACHPt)_16_/5-FU and G2.5(COONa)_32_/5-FU were characterized by NMR (^1^H, ^13^C, and ^19^F). The G2.5COO(DACHPt)_16_/5-FU has an upfield shift of the protons in the ^1^H-NMR spectrum (Figure S28). The characteristic protons of 5-FU are visible in the spectrum after encapsulation. For the ^13^C-NMR spectrum, a shift is observed for carbons bonded just after to the amide groups inside the metallodendrimer, and carbons bonded after the tertiary amine groups, from 48.63 ppm to 48.86 ppm (Figure S29). The carbon signals of the amide groups inside the metallodendrimer also shift from 173.68 ppm to 173.08 ppm. Regarding the 5-FU carbon signals, the only signal visible in the spectrum is the signal related to the C-6 carbon, meaning that the carbonyl groups and amide groups are involved in the interaction with the metallodendrimer. The ^19^F-NMR spectrum (Figure S30) reveals a non-significant shift (0.08 ppm), probably because the fluorine is not involved in the interaction with the metallodendrimer. Based on the previous results, we suggest that 5-FU molecules should be bonded through the amide and tertiary amine groups of the metallodendrimers. In the ^1^H spectrum of G2.5(COONa)_32_/5-FU (Figure S31), it is possible to observe a downfield shift in all the characteristic signals of the G2.5(COONa)_32_ PAMAM dendrimer after encapsulated 5-FU molecules, which is prominent in the signals of methylene protons present at tertiary amine groups and of the amide groups. Even though it is not so significant, this evidence is also perceptible in the ^13^C spectrum (Figure S32), particularly in the carbons bonded after the tertiary amine group (from 49.78 to 50.13 ppm) located in the complex’s interior and also in the carbon bonded after the amide group located just below to the surface (from 34.9 ppm to 34.2 ppm). The corresponding signal of the amide carbons located inside the dendrimer shifts from 178.82 ppm to 177.57 ppm, corroborating the presence of 5-FU inside the dendrimer and not attached to its surface. Moreover, the signal of the carbon from the carboxylic end groups does not shift. Regarding 5-FU, in general, a slight shift on the carbons was observed, with the signal of carbon C-2 absent after encapsulation and the C-4 carbon presenting a significant shift (from 159.74 ppm to 156.78 ppm), suggesting that the carbonyl group is protonated, and this is the main group that interacts with the PAMAM dendrimer. Besides, the fluorine signal in the ^19^F-NMR spectrum is shifted downfield (Figure S33), indicating that it is also involved in the interaction with the PAMAM dendrimer.

#### 2.4.2. Cytotoxicity of the Complex

A2780CisR and CACO-2 cancer cell lines were used to evaluate the cytotoxicity of the G2.5COO(DACHPt)_16_/5-FU system and were selected because 5-FU is used in the treatment of colorectal cancer, but also in the cases of other cancer types, such as ovarian cancer [49,50,51]. The IC_50_ results are presented in Table 3. From the IC_50_ values, we observe that the G2.5COO(DACHPt)_16_/5-FU complex, compared to free 5-FU and G2.5(COONa)_32_/5-FU, has an IC_50_ much lower in both cancer cell lines, which is due to the presence of the metallic fragments in the dendrimers that should be released along time and exert their anticancer activity. When using the A2780cisR cells, the IC_50_ value decreases from 1.1 μM to 0.2 μM for the non-loaded and the loaded metallodendrimer, respectively. However, when using CACO-2 cells, the IC_50_ slightly increases when 5-FU molecules are loaded into the metallodendrimer. As such, it seems that the metallic fragment-induced cytotoxicity dominates over the cytotoxicity of 5-FU. This is evident when we compare the IC_50_ values for the free drugs oxaliplatin and 5-FU as IC_50_ (oxaliplatin) << IC_50_ (5-FU). In vivo, however, one should expect beneficial effects of the co-delivery of DACHPt and 5-FU, as happens when combined therapy is used in the patients.

#### 2.4.3. Hemotoxicity of the Complex

**Figure 8:**
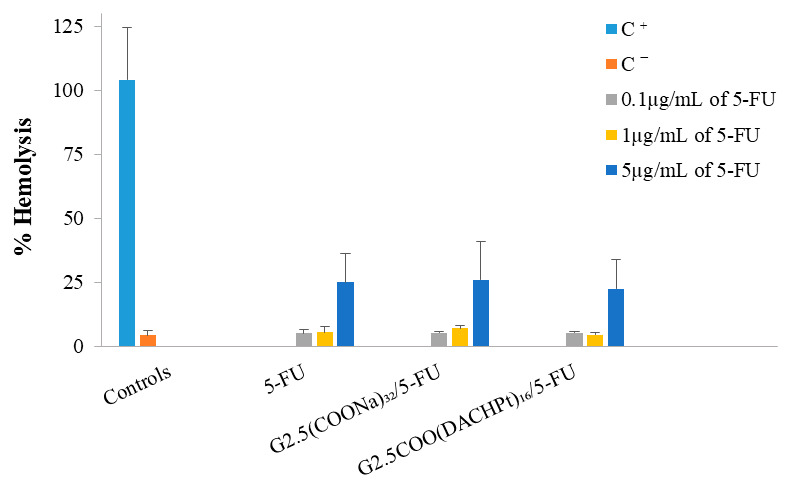
Hemotoxicity of the free 5-FU, G2.5(COONa)_32_/5-FU, and G2.5COO(DACHPt)_16_/5-FU. Blood was treated for 3 h with different concentrations (0.1, 1, and 5 µg/mL) of the complexes and free 5-FU. The positive and negative control are represented by C^+^ and C^−^, respectively. The results are expressed as mean ± SD of at least three independent experiments performed in triplicate.

The results of hemolysis (Figure 8) show that the G2.5COO(DACHPt)_16_/5-FU system and the 5-FU are not hemotoxic at concentrations 0.1 and 1 µg/mL, only achieving ca. 5% of hemolysis. Nonetheless, when we increase the concentration to 5 µg/mL, the percentage of hemolysis significantly increases to a value between 24–26% for all the compounds considered, which should be attributed to 5-FU presence.

#### 2.4.4. In Vitro Drug Release

**Figure 9:**
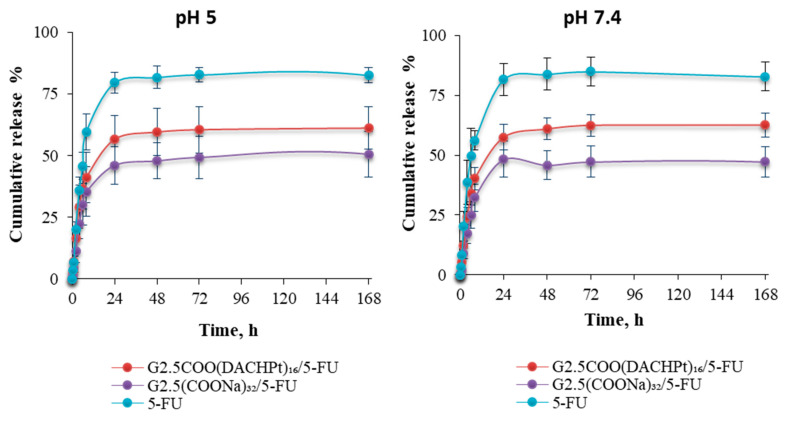
The release profile of 5-FU from DACHPt metallodendrimer and anionic PAMAM dendrimer in pH 5 and 7.4 at 37 °C.

From Figure 9, we can conclude that, after an initial faster release of the 5-FU drug from G2.5COO(DACHPt)_16_/5-FU and G2.5COO(COONa)_32_/5-FU systems, drug release starts to be sustained along time, achieving maximum values of 57% and 46% of cumulative release after 24 h, respectively. That is, drug release seems to be higher for the metallodendrimer than for the G2.5(COONa)_32_ dendrimer. On the other end, decreasing the pH value does not affect drug release, which means that our system is not sensitive to the pH, which, as it is known, may change towards more acidic values in the microtumor environment.

## 3. Materials and Methods

### 3.1. Materials

Anionic PAMAM dendrimer half-generations, G0.5 (19.19 *w*/*w* % in methanol), G1.5 (20.03 *w*/*w* % in methanol), G2.5 (9.98% *w*/*w* in methanol) or G2.5 (3.43 *w*/*w* % in water), and G3.5 (10.04 *w*/*w* % in methanol) with an ethylenediamine core were purchased from Dendritech^®^ Inc. (Midland, MI, USA) and purified by dialysis before its use to eliminate further impurities. *trans*-(R,R)-1,2-diaminocyclohexane (99%) and [(1R,2R)-cyclohexane-1,2-diamine] (ethanedioato-O,O′)platinum(II) (oxaliplatin 99%) were bought from Acros Organics (Geel, Antwerp, Belgium), and 5-fluoro-1H-pyrimidine-2,4-dione (5-fluorouracil, 99%) from TCI chemicals (Zwijndrecht, Belgium). Deoxyribonucleic acid sodium salt from calf thymus (CT-DNA) and 4,6-diamino-2-phenyindole dilactate (DAPI) were obtained from Sigma-Aldrich (St. Louis, MO, USA). The remaining reagents were acquired from Acros Organics and Fisher Scientific (Lisboa, Portugal) and used without further purification. The ultrapure water (UPW) was obtained with a Milli-Q Direct 8 Water Purification System with a resistivity higher than 18.2 MΩ·cm. All the media, solutions, and reagents used for cell culture manipulation were purchased from Life Technologies (Thermo Fischer Scientific, London, UK) unless otherwise stated. The healthy human blood was supplied by Hospital Dr. Nélio Mendonça (SESARAM) under a collaboration between the University of Madeira/Centro de Química da Madeira and the SESARAM hematology service.

### 3.2. Synthesis and Characterization

The methodology used for the preparation of *trans*-(R,R)-1,2- diaminocyclohexanedichloroplatinum(II) (DACHPtCl_2_) was adapted from the patent USOO8637692B2 [52]. The synthesis of DACHPt metallodendrimers was based on the procedure described by G. J. Kirkpatrick et al. [40], H. Nguyen et al. [53], and N. Q. Tran et al. [54]. The NMR characterization was performed in Bruker Avance II+ UltraShield^TM^ 400 Plus Ultra Long Hold NMR spectrometer equipment at room temperature. The mass spectrometry (MS) analysis was performed using the MALDI ionization technique with ULTRAFLEX III TOF/TOF equipment from Bruker (Leipzig, Germany) and ESI, and the electrospray ionization technique, in positive mode, was performed by the QTOF hybrid analyzer model MAXIS II from Bruker (Leipzig, Germany) at the Mass Spectrometry Unit at the Interdepartmental Investigation Service at Universidad Autónoma de Madrid, Spain. Accordingly, for G0.5COO(DACHPt)_4_ and G3.5COO(DACHPt)_32_ metallodendrimers, MALDI was used with the matrix, α-cyano-4-hydroxycinnamic acid (ACC). However, in the metallodendrimer G3.5COO(DACHPt)_32_, MALDI was used in the reflector mode. The metallodendrimers G1.5COO(DACHPt)_8_ and G2.5COO(DACHPt)_16_ were characterized by ESI in the positive mode. For G1.5COO(DACHPt)_8_, a direct infusion of a 1:10 dilution in methanol with 0.1% formic acid of the initial sample solution was performed, and the G2.5COO(DACHPt)_16_ was performed in the exact mass with methanol and 0.1% formic acid as ionizing phase. FTIR analysis was made with a PerkinElmer Spectrum Two spectrometer apparatus, and a PerkinElmer UV-vis spectrometer Lambda was used for UV-vis studies. Fluorescence studies were conducted in PerkinElmer LS 55 fluorescence spectrometer equipment (Perkin-Elmer, Waltham, MA, USA. The zeta-potential measurements were performed using a Zetasizer Nano ZS equipment (Malvern Instruments Ltd., Malvern, UK), with the compounds being dissolved in ultrapure water (UPW) with a concentration of 0.3 mg/mL. Three independent experiments were obtained for each sample. The UPW used was filtered before its use with a filter of 0.2 µm.

#### 3.2.1. Preparation of DACHPtCl_2_

For the preparation of DACHPtCl_2_, 0.35 g of potassium tetrachloroplatinate (0.84 mmol) was dissolved in 17.5 mL of UPW. Then, 0.1 g of *trans*-(R,R)-1,2-diaminocyclohexane (0.87 mmol; 1.03 eq. mol) in 5.5 mL of UPW was added dropwise to the mixture under stirring. The reaction was left for 7 h at r.t. in the dark (Scheme S1). A change in the color of the solution from red to toasted yellow was observed (yellow suspension). The yellow precipitate DACHPtCl_2_ was filtrated through a 0.22 µm nylon filter, washed with 40 mL of distilled water, 30 mL of methanol, and acetone, and dried in vacuum for 1 h. A yellow powder was obtained with a 59% of yield (0.19 g). ^1^H-NMR (400 MHz, D_2_O) (ppm): δ = 1.13 (3,3′α-H; m, 2H), 1.26 (2,2′ α -H; m, 2H), 1.55 (3,3′ β-H; d, 2H), 2.01 (2,2′ β-H; d, 2H), 2.40 (1,1′; m, 2H) and 3.39 (NH, broad, 4H). FTIR: v = 3186 cm^−1^ (N–H amine), 3276 cm^−1^ (N–H amine), 1566 cm^−1^ (N–H amine), 2865 cm^−1^ (C–H), 2935 cm^−1^ (C–H). UV-vis: 212 nm maximum absorption wavelength. Fluorescence: λ_em,max_ = 429 nm (for λ_ex_ = 380 nm, in UPW).

#### 3.2.2. Aquation of DACHPtCl_2_

In the aquation process, silver nitrate was used to remove both chloride ligands from the DACHPtCl_2_. As such, 0.18 g of DACHPtCl_2_ (0.48 mmol) was dispersed in 100 mL of UPW. Then, an aqueous solution of 0.16 g of AgNO_3_ (0.95 mmol, 2 equiv. mol) in 6.5 mL of UPW was added dropwise to the mixture under stirring. Then, the solution was left under nitrogen atmosphere and stirring for 24 h at r.t. protected from light (Scheme S2). After that time, a “milky-white” precipitate (silver chloride precipitate) was observed, indicating the formation of the DACHPt(H_2_O)_2_ complex. The silver chloride precipitate was removed by centrifugation at 15,000 rpm for 1.5 h at 25 °C. The remaining supernatant was filtrated through a 0.22 μm nylon filter and freeze-dried for 3 days. In the end, a sand color powder was obtained with 86% yield (0.14 g). ^1^H-NMR (400 MHz, D_2_O) (ppm): δ = 1.14 (3,3′α-H; m, 2H), 1.30 (2,2′ α -H; m, 2H), 1.56 (3,3′ β-H; d, 2H), 2.03 (2,2′ β-H; d, 2H) and 2.39 (1,1′; m, 2H). 13C-NMR (100 MHz, D_2_O) (ppm): δ = 23.22 (3,3′), 30.66 (2, 2′) and 62.39 (1,1′).

#### 3.2.3. Preparation of DACHPt Metallodendrimers

##### Anionic PAMAM Dendrimer Generation 0.5-G0.5COO(DACHPt)_4_

The DACHPt(H_2_O)_2_ complex (0.086 g, 0.25 mmol, 4.5 eq. mol) was dispersed in 35 mL of UPW. Then, 0.07 g (0.06 mmol) of G0.5COONa PAMAM dendrimer was dissolved in 5 mL of UPW and added dropwise to the mixture under stirring. The mixture was left to react for 24 h at r.t. in the dark under nitrogen atmosphere (Scheme S3). The resulting solution was purified using a dialysis membrane (MW 500–1000 Da) for 7 h in distilled water. After being freeze-dried, a “greenish-yellow” hygroscopic powder was obtained with 92% yield (0.12 g). ^1^H-NMR (400 MHz, D_2_O) (ppm): δ = 1.18 (3,3′α-H; m, 12H), 1.30 (2,2′ α -H; m, 8H), 1.61 (3,3′ β-H; d, 10H), 2.05 (2,2′ β-H; d, 10H), 2.43 (1,1′; m, 10H), 2.63 (Hc + Hi, m, 25H), 2.97 (Ha + Hg, m, 13H), 3.24 (Hb, 8H), 3.31 (Hh, 16H) and 3.61 (Hf, 8H). ^13^C-NMR (100 MHz, D_2_O) (ppm): δ = 23.32 (3,3′), 30.73 (Cc), 31.01 (2, 2′), 31.40 (Ci), 34.35 (Cf), 48.61 (Ca + Cg), 50.27 (Ch), 50.94 (Cb), 62.02 (1,1′) and 177.83 (Cp). ^195^Pt-NMR (86 MHz, D_2_O) (ppm): δ = −2313.58. FTIR (KBr pellet): v = 1584 cm^−1^ (amide II, N–H and C–N), 1618 cm^−1^ (C=O), 2890 cm^−1^ (C–H), 2934 cm^−1^ (C–H), 3231 cm^−1^ (N–H stretch of the amine group of DACHPt) and 3469 cm^−1^ (N–H). Fluorescence: λ_em,max_ = 457 nm (for λ_ex_ = 380 nm, in UPW). TOF-MS (MALDI) = *m*/*z* calc. = 2014.75, *m*/*z* found = 2014.70 [M + H^+^] C_64_H_117_N_16_O_20_Pt^3+^.

##### Anionic PAMAM Dendrimer Generation 1.5-G1.5COO(DACHPt)_8_

The DACHPt(H_2_O)_2_ complex (0.05 g, 0.15 mmol, 8.5 eq. mol) was dispersed in 27 mL of UPW. Then, 0.05 g (0.02 mmol) of G1.5COONa PAMAM dendrimer was dissolved in 6 mL of UPW and added dropwise to the solution under stirring. The mixture was left to react for 30 h at r.t. in the dark under nitrogen atmosphere (Scheme S4). The resulting solution was then purified using a dialysis membrane (MW 2 KDa) for 6 h in distilled water and freeze-dried. A hygroscopic grey powder was obtained with 77% yield (0.07 g). ^1^H-NMR (400 MHz, D_2_O) (ppm): δ = 1.20 (3,3′α-H; m, 22H), 1.32 (2,2′ α -H; m, 16H), 1.61 (3,3′ β-H; d, 18H), 2.07 (2,2′ β-H; d, 20H), 2.47 (1,1′; m, 21H), 2.52 (Hc + Hi, m, 38H), 2.62 (Ho, 36H), 2.72 (Ha + Hg, 26H), 2.94 (Hn, 48H) and 3.30 (Hm, 28H), 3.35 (Hb + Hh, m, 44H) and 3.63 (Hf + Hl, m, 16H). ^13^C-NMR (100 MHz, D_2_O) (ppm): δ = 23.47 (3,3′), 30.38 (Co), 31.06 (2, 2′), 31.66 (Ci + Cc), 34.24 (Cf + Cl), 48.52 (Cb + Ch), 50.33 (Cn), 50.98 (Ca + Cg + Cm), 61.92 (1,1′), 174.68 (Cd) and 177.55 (Cp). ^195^Pt-NMR (86MHz, D_2_O) (ppm): δ = −2321.68. FTIR: v = 1582 cm^−1^ (amide II, N–H and C–N), 1639 cm^−1^ (C=O), 2859 cm^−1^ (C–H), 2939 cm^−1^ (C–H), 3278 cm−^1^ (N–H stretch of the amine group of DACHPt) and 3460 cm^−1^ (N–H). Fluorescence: λ_em,max_ = 454 nm (for λ_ex_ = 380 nm, in UPW). TOF-MS (ESI+) = *m*/*z* calc. = 1008.58, *m*/*z* found = 1008.98 [M + 5H^+^] C_158_H_293_N_42_O_44_Pt_8_^5+^.

##### Anionic PAMAM Dendrimer Generation 2.5-G2.5COO(DACHPt)_16_

The DACHPt(H_2_O)_2_ complex (0.1 g, 0.29 mmol, 16.5 eq. mol) was dispersed in 41 mL of UPW. Then, 0.11 g (0.02 mmol) of G2.5COONa PAMAM dendrimer was dissolved in 10 mL of UPW and added dropwise to the mixture under stirring. The reaction mixture was left to react for 40 h at r.t. in the dark under nitrogen atmosphere (Scheme S5). Then, the mixture was purified using a dialysis membrane (MW 3.5 KDa) for 6 h in distilled water and freeze-dried. A dark brown hygroscopic solid was obtained with 78% yield (0.14 g). ^1^H-NMR (400 MHz, D_2_O) (ppm): δ = 1.20 (3,3′α-H; m, 23H), 1.34 (2,2′ α -H; m, 20H), 1.61 (3,3′ β-H; d, 20H), 2.07 (2,2′ β-H; d, 20H), 2.38 (1,1′; m, 12H), 2.65 (Hc + Hi + Ho + Hu, m, 146H), 3.00 (Ha + Hm + Hg + Hs, m, 92H), 3.21 (Hf + Hl, 23H), 3.40 (Hn + Hb + Hh + Hn, m, 131H) and 3.67 (Hr, 32H). ^13^C-NMR (100 MHz, D_2_O) (ppm): δ = 23.40 (3,3′), 29.93 (Cu), 30.97 (Cc + Ci + Co), 31.49 (2, 2′), 34.02 (Cr), 48.63 (Cb + Cf + Cl + Ch + Cn), 50.31 (Ct), 51.02 (Ca + Cg + Cm + Cs), 61.84 (1,1′), 173.68 (Cd) and 177.17 (Cp). ^195^Pt-NMR (86 MHz, D_2_O) (ppm): δ = −2316.89. FTIR (KBr pellet): v = 1584 cm^−1^ (amide II, N–H and C–N), 1640 cm^−1^ (C=O), 2855 cm^−1^ (C–H), 2939 cm^−1^ (C–H), 3224 cm^−1^ (N–H stretch of the amine group of DACHPt) and 3410 cm^−1^ (N–H). Fluorescence: λ_em,max_ = 457 nm (for λ_ex_ = 380 nm, in UPW).TOF-MS (ESI+) = *m*/*z* calc. = 1558.71, *m*/*z* found = 1556.70 [M + H^+^+ 8MeOH]^+^ C_342_H_641_N_90_O_100_Pt_16_^+^.

##### Anionic PAMAM Dendrimer Generation 3.5-G3.5COO(DACHPt)_32_

The DACHPt(H_2_O)_2_ complex (0.13 g, 0.38 mmol, 32.5 eq. mol) was dispersed in 53 mL of UPW. 0.15 g (0.01 mmol) of G3.5COONa PAMAM dendrimer was dissolved in 13 mL UPW and added dropwise to the mixture under stirring. The mixture was left to react for 43 h at r.t. in the dark under nitrogen atmosphere (Scheme S6). After, the resulting solution was purified using a dialysis membrane (MW 6–8 KDa) for 6 h in distilled water. After being freeze-dried, a dark brown hygroscopic solid was obtained with 60% yield (0.15 g). ^1^H-NMR (400MHz, D_2_O) (ppm): δ = 1.19 (3,3′α-H; m, 27H), 1.34 (2,2′ α -H; m, 27H), 1.61 (3,3′ β-H; d, 26H), 2.07 (2,2′ β-H; d, 26H), 2.48 (1,1′; m, 22H), 2.59 (Hc + Hi + Ho + Hu, m, 84H), 2.65 (HҰ, t, 164H), 3.08 (Ha + Hm + Hg + Hs, 162H), 3.26 (Hy, t, 71H), 3.44–3.38 (Hz + Hn + Hb + Hh + Hn, m, 262H), 3.59 (Hl + Hf + Hr, t, 40H) and 3.69 (Hx, 64H). ^13^C-NMR (100 MHz, D_2_O) (ppm): δ = 23.40 (3,3′), 29.88 (Cұ), 31.56 (Co + Ci + Cc + Cu), 32.77 (2,2′), 34.00 (Cx), 35.39 (Cl + Cf + Cr), 48.65 (Ca + Cg + Cm+ Cs + Cy), 50.29 (Cz), 51.07 (Cb + Ch + Cn + Ct), 60.09 (1,1′), 173.21 (Cd) and 177.11 (Cp). ^195^Pt-NMR (86 MHz, D_2_O) (ppm): δ = −2312.63. FTIR (KBr pellet): v = 1588 cm^−1^ (amide II, N–H and C–N), 1638 cm^−1^ (C=O), 2855 cm^−1^ (C–H), 2937 cm^−1^ (C–H), 3244 cm^−1^ (N–H stretch of the amine group of DACHPt) and 3417 cm^−1^ (N–H). Fluorescence: λ_em,max_ = 452 nm (for λ_ex_ = 380 nm, in UPW).TOF-MS (MALDI) = *m*/*z* calc. = 388.9, *m*/*z* found = 388.2 [M] C_632_H_1140_N_168_O_188_Pt_23_.

### 3.3. DNA Binding Studies by UV–Vis Spectroscopy

Absorption spectra were performed at room temperature for varying concentrations of CT-DNA (0, 6.25, 12.5, 18.75, 25, 31.25, 37.5, 43.75 to 50 µM) and constant concentrations of G2.5COO(DACHPt)_16_ metallodendrimer (2 µM), DACHPtCl_2_ (9 µM), or oxaliplatin (9 µM). The metallodendrimer, DACHPtCl_2_, and oxaliplatin solutions were prepared in ultrapure water and then diluted in a 5 mM Tris-HCl, 50 mM NaCl pH 7.4 buffer. The stock solutions of DNA were directly prepared in this buffer. DNA purity was assessed by UV–vis spectroscopy using the absorbance values ratio at 260 nm and 280 nm (it should be between 1.8–1.9 to make sure it is sufficiently protein-free). The obtained ratio was 1.9, indicating that the DNA was pure. The compounds and the CT-DNA were incubated for 5 min at room temperature. The absorbance was measured in a PerkinElmer UV–vis spectrometer Lambda equipment, using the buffer as blank. Two independent experiments were carried out for the metallodendrimers, DACHPtCl_2,_ and oxaliplatin. The intrinsic binding constant, K_b_, of the compounds with DNA, was determined using the Benesi–Hildebrand equation [36]: A_0_/(A−A_0_) = ε_G_/(ε_H-G_ − ε_G_) + ε_G_/(ε_H-G_ − ε_G_) × 1/(K_b_[DNA]), where K_b_ is the binding constant, [DNA] is the DNA concentration, A_0_ and A are the absorbance values of the free compound and compound–DNA complex, and ε_G_ and ε_H-G_ are their absorption coefficients, respectively. Binding constant values were then obtained from the ratio of the y-intercept to the slope in the plots A_0_/A−A_0_ vs. 1/[DNA]. Additionally, the Gibbs free energy (ΔG) associated with the process of DNA binding was calculated using the usual equation: ∆G = −RTlnK_b_, where T is the temperature in Kelvin and R the gas constant.

### 3.4. Cell Culture and Cytotoxicity Evaluation

Several human cell lines were used in the cytotoxicity evaluation studies, namely: ovarian cancer cells (A2780), cisplatin-resistant ovarian cancer cells (A2780CisR), breast cancer cells (MCF-7), colorectal adenocarcinoma cells (CACO-2), and fibroblast cells (BJ, a non-cancer cell line). All cell lines were cultured in 96-well plates at a seeding density of 1 × 10^4^ cells per well with a specific culture medium supplemented with 10% (*v*/*v*) fetal bovine serum (FBS) and 1% (*v*/*v*) antibiotic–antimycotic solution (AA, 100× solution) at 37 °C, in a humidified atmosphere and 5% CO_2_. Specific cell culture media were as follows: the A2780 and A2780CisR cell lines were cultured in RPMI 1640 medium supplemented with L-glutamine (2 mM), and 1% (*w*/*v*) of cisplatin (100 mM) in the case of A2780CisR cells (cisplatin was only used in the subculturing process before the cytotoxicity assays); the MCF-7 cell line was cultured in RPMI 1640 medium supplemented with 1 mM sodium pyruvate, 1% (*v*/*v*) nonessential amino acids (NEAA, 100× solution), and human insulin 3.3 µg/mL; the CACO-2 cell line was cultured in MEM medium supplemented with 1% (*v*/*v*) NEAA; the BJ cell line was cultured in D-MEM medium. After 24 h in culture, cells were incubated with the compounds under testing prepared in nuclease-free water. In all cases, 100 µL of the compound solution was used for a total volume of 200 µL in the well. The metallodendrimers cytotoxicity was evaluated using all the mentioned cell lines at the concentrations of 0.01, 0.03, 0.1, 0.5, 1, 2.5, 5, and 10 µM. Pristine anionic PAMAM dendrimers (G0.5–3.5) were used as controls. The cytotoxicity of the metallodendrimer G2.5COO(DACHPt)_16_/5-FU was evaluated using A2780CisR, and CACO-2 cells at the concentrations of 0.01, 0.2, 1, 5, 10 and 20 µg/mL, and G2.5(COONa)_32_/5-FU and free 5-FU were used as controls. After 72 h of incubation with the compounds under testing, the culture medium was replaced by new culture medium containing 10% (*v*/*v*) of a MTT (3-(4,5-dimethylthiazol-2-yl)-2,5-diphenyltetrazolium bromide) solution (0.5 mg/mL). After 3 h of incubation, the culture medium was aspirated, and the formed formazan crystals were dissolved in 100 µL of DMSO. Absorbance intensity was measured at 550 nm in a microplate reader (Victor3 1420, Perkin Elmer, Waltham, MA, USA). Three independent experiments with three replicas each were carried out. The concentration that inhibited 50% of the cellular metabolic activity (IC_50_) was determined by linear interpolation between the two experimental points closer to the point correspondent to 50% of the control’s cellular metabolic activity. Results are presented as the mean ± standard deviation (SD).

### 3.5. Hemotoxicity Evaluation

Human blood from healthy donors was collected in tubes containing EDTA and used to evaluate the compounds’ hemotoxicity under testing towards red blood cells by the cyanmethemoglobin method [55]. The total hemoglobin concentration in the original blood was determined starting from a 250-fold dilution of blood in cyanmethemoglobin reagent (the C reagent was prepared in an amber bottle with 50 mg of potassium ferricyanide, 12.5 mg of potassium cyanide, and 35 mg of potassium dihydrogen phosphate in 250 mL of distilled water with 250 µL of Triton-X; its pH was adjusted to 7.4). A standard curve for hemoglobin was then prepared with hemoglobin from bovine blood (Figure S34). Briefly, a stock solution of the protein (1.5 mg/mL) was first prepared in C reagent, from which serial dilutions were performed to obtain standards of known concentration (in the range 0.20 to 1.4 mg/mL). Absorbance was then measured at 550 nm, and the C reagent was used as blank. The total hemoglobin concentration was determined using this standard curve, taking into consideration the initial dilution. For hemotoxicity evaluation of the compounds under testing, a 10% (*v*/*v*) blood solution was prepared in PBS (Mg^2+^/Ca^2+^ free). Then, 10 µL of this blood solution was added to several microtubes containing 70 µL of compound solution (at the concentrations 0.1, 1, and 5 µM); for controls, microtubes with 70 µL of distilled water (positive control) and 70 µL of PBS (negative control) were also prepared. After, the microtubes were incubated at 37 °C for 3 h and subsequently centrifuged at 3800 rpm for 10 min. In the end, 40 µL of each supernatant were transferred to 96-well plates, 160 µL of C reagent was added, and absorbance was measured at 550 nm. The concentration of hemoglobin in the supernatants was then determined using the same standard curve and considering the performed dilutions. The results are presented as a percentage of hemolysis (mean of three independent assays) ± standard deviation (SD). A Perkin Elmer VICTOR^3^™ Multilabel Reader spectrophotometer was used in this assay.

### 3.6. Studies with 5-Fluorouracil Loaded Dendrimers

#### 3.6.1. Loading of 5-FU

G2.5COO(DACHPt)_16_ metallodendrimers were loaded with 5-Fluorouracil. For that purpose, 25 mg (0.002 mmol) of G2.5COO(DACHPt)_16_ was dissolved in 2 mL of UPW, and 4.7 mg of 5-FU (15 eq. mol, 0.04 mmol) was added to the solution. As a control, the G2.5(COONa)_32_ dendrimer (25 mg, 0.004 mmol, 2 mL) was also loaded with 5-FU (7.8 mg, 0.06 mmol) in similar conditions. Then, the G2.5COO(DACHPt)_16_/5-FU and the G2.5(COONa)_32_/5-FU solutions were dialyzed in 50 mL of distilled water using a dialysis membrane in the MW range of 100–500 Da for 20 min to remove the unloaded 5-FU. Later, the G2.5COO(DACHPt)_16_/5-FU and G2.5(COONa)_32_/5-FU solutions and the solution outside the dialysis membrane were lyophilized. The free drug was dissolved in 50 or 60 mL of UPW, and its absorbance was measured at 266 nm in PerkinElmer UV–vis spectrometer Lambda equipment to determine the amount of drug loaded into both dendrimer types indirectly. For the quantification of 5-FU in solution, a standard calibration curve was first established using standards of the known concentration of 5-FU in water (Figure S35). The loading capacity (LC%) and the loading efficiency (LE%) were calculated through the following formulas:

LC (%) = (Mass of loaded 5-FU/(Mass of loaded 5-FU + Mass of dendrimer)) × 100(1)

LE (%) = ((Initial mass of 5-FU − Mass of free 5-FU)/Initial mass of 5-FU) × 100(2)

The results are expressed as mean ± SD of three independent experiments.

#### 3.6.2. In Vitro Drug Release of 5-FU

The release of 5-FU was made in PBS in acid conditions (pH adjusted to 5) and at physiologic pH (pH adjusted to 7.4), at 37 °C. For this aim, 100 µg of 5-FU loaded in G2.5COO(DACHPt)_16_ and to G2.5(COONa)_32_ were weighted and dissolved in 300 µL of water. The solutions were placed in a SLIDE-A-LYZER™ mini dialysis device with an MW cutoff of 2 KDa (0.1 mL, Thermo Fisher Scientific, London, UK) and dialyzed in 10 mL of PBS, in separate tubes, at each pH value. At different time intervals, 1 mL of the dialyzed solution was taken out from each tube and replaced with an equivalent volume of fresh PBS. The release profile of 5-FU was then determined by UV–vis spectroscopy. Standard calibration curves were established for each PBS pH value (Figures S36 and S37). Absorbance was measured at 266 nm with a PerkinElmer UV–vis spectrometer Lambda (Waltham, MA, USA).

## 4. Conclusions

Half-generation PAMAM dendrimers (G0.5–G3.5) with carboxylate end-groups were used as nanocarriers of the active metallic fragment DACHPt to take advantage of its good anticancer activity and to overcome some of the associated problems of the drug DACHPtCl_2_, such as its low water solubility. The prepared DACHPt metallodendrimers showed an in vitro cytotoxic effectiveness higher than oxaliplatin in several cell lines, as well as low hemotoxicity. DNA binding studies in the presence of increasing CT-DNA concentrations revealed a hyperchromic effect, indicative of a disruption of the DNA double helix. The prepared DACHPt metallodendrimers were also shown to be able to load and release the anticancer drug 5-FU, although the cytotoxicity of the metallic fragment DACHPt dominated over 5-FU cytotoxicity. In conclusion, promising results on the use of DACHPt metallodendrimers alone or combined with 5-FU were obtained in vitro, and further studies, including in vivo experiments, should be carried out in the future to confirm the full potential of these systems. Indeed, we believe that it will be possible to optimize the DACHPt metallodendrimer/5-FU systems (preparation, scale-up, and integration of a third drug) to act as an alternative for the current FOLFOX chemotherapy regimen used to treat stage III colorectal cancer and its recurrences.

## Figures and Tables

**Table 1:** Zeta-potential of anionic PAMAM dendrimers (G0.5–G3.5) and their related metallodendrimers after coordination with DACHPt.

Compounds	Zeta-Potential (mV)
G0.5(COONa)_8_	−19 ± 1
G0.5COO(DACHPt)_4_	−2.3 ± 0.5
G1.5(COONa)_16_	−40.8 ± 0.7
G1.5COO(DACHPt)_8_	−17 ± 2
G2.5(COONa)_32_	−48 ± 1
G2.5COO(DACHPt)_16_	−10.8 ± 0.3
G3.5(COONa)_64_	−51 ± 1
G3.5COO(DACHPt)_32_	4.0 ± 0.6

**Table 2:** Values of DNA binding constant (K_b_) and Gibbs free energy (ΔG) for the G2.5(COO(DACHPt)_16_ metallodendrimer and the free drugs, DACHPtCl_2_ and oxaliplatin. Data are represented as mean ± SD of two independent experiments.

Compounds	Change in Absorbance	K_b_ (M^−1^)	−ΔG/KJ mol^−1^
G2.5COO(DACHPt)_16_	Hyperchromism	(3.6 ± 0.9) × 10^4^	0.25 ± 0.01
DACHPtCl_2_	Hyperchromism	(3 ± 1) × 10^3^	0.19 ± 0.01
Oxaliplatin	Hyperchromism	(3.1 ± 0.6) × 10^3^	0.19 ± 0.01

**Table 3:** IC_50_ values of the prepared DACHPt metallodendrimers and DACHPt metallodendrimers with 5-FU (Section 2.4.2) towards various cancer cell lines and a non-cancer cell line. Results are expressed as mean ± SD of three independent experiments performed in triplicate.

Compounds	A2780 IC_50_ ± SD (µM)	A2780cisR IC_50_ ± SD (µM)	MCF-7 IC_50_ ± SD (µM)	CACO-2 IC_50_ ± SD (µM)	BJ IC_50_ ± SD (µM)
Oxaliplatin	0.48 ± 0.03	3.5 ± 0.5	>10	0.91 ± 0.03	>10
DACHPtCl_2_	0.3 ± 0.2	1.7 ± 0.4	5 ± 2	>10	>9
G0.5COO(DACHPt)_4_	0.03 ± 0.01	1.7 ± 0.3	1.6 ± 0.8	0.18 ± 0.08	3 ± 1
G1.5COO(DACHPt)_8_	0.04 ± 0.02	0.6 ± 0.2	1.6 ± 0.7	0.3 ± 0.1	1.3 ± 0.2
G2.5COO(DACHPt)_16_	0.04 ± 0.03	1.1 ± 0.2	3 ± 1	0.35 ± 0.09	1.8 ± 0.7
G3.5COO(DACHPt)_32_	0.08 ± 0.02	1.2 ± 0.5	4.1 ± 0.8	0.39 ± 0.09	3 ± 1
5-FU	−	>154	–	>154	–
G2.5COO(DACHPt)_16_/5FU *	−	0.2 ± 0.1	–	0.65 ± 0.06	–
G2.5(COONa)_32_/5-FU *	−	>2.5	–	>2.5	–

* For the calculation of the MW, the estimated number of 5-FU molecules carried by the dendrimer was taken into account.

**Table 4:** Relative potency (RP) of the DACHPt metallodendrimers calculated from the division of the IC_50_ oxaliplatin value by the IC_50_ metallodendrimers value.

	Relative Potency (RP)			
Compounds	A2780	A2780CisR	MCF-7	CACO-2
DACHPtCl_2_	1.7	2	>1.9	>0.1
G0.5COO(DACHPt)_4_	16	2.1	>6.4	5
G1.5COO(DACHPt)_8_	12	5.9	>6.3	3.6
G2.5COO(DACHPt)_16_	12	3.2	>3.4	2.6
G3.5COO(DACHPt)_32_	6	2.9	>2.4	2.3

**Table 5:** Selectivity index (SI) of the DACHPt metallodendrimers calculated from the division of the IC_50_ BJ cell line value by the IC_50_ cancer cell lines value.

	Selectivity Index (SI)			
Compounds	A2780	A2780CisR	MCF-7	CACO-2
Oxaliplatin	>20.8	>2.9	>1	>11
DACHPtCl_2_	>32	>5.2	>1.7	>0.9
G0.5COO(DACHPt)_4_	103	1.9	2	17
G1.5COO(DACHPt)_8_	31	2	0.8	5
G2.5COO(DACHPt)_16_	46	1.7	0.6	5
G3.5COO(DACHPt)_32_	32	2	0.6	6.6

**Table 6:** Resistance factor of the DACHPt metallodendrimers calculated from the division of the IC_50_ A2780CisR value by the IC_50_ A2780 cancer cell lines value.

Compounds	Resistance Factor (Rf)
Oxaliplatin	7.2
DACHPtCl_2_	6.2
G0.5COO(DACHPt)_4_	55.3
G1.5COO(DACHPt)_8_	14.8
G2.5COO(DACHPt)_16_	27
G3.5COO(DACHPt)_32_	15

**Table 7:** Loading efficiency (LE%) and loading capacity (LC%) of 5-FU in G2.5COO(DACHPt)_16_ metallodendrimer and in the anionic PAMAM dendrimer G2.5COONa (*n* = 3). The corresponding number of encapsulated 5-FU molecules is shown.

Compounds	LE%	LC%	N° of Encapsulated Molecules ^1^
G2.5COO(DACHPt)_16_/5-FU	75 ± 8	14 ± 1	11
G2.5(COONa)_32_/5-FU	86 ± 2	32 ± 1	13

^1^ The number of encapsulated molecules was calculated from the following equation, i (number of encapsulated molecules) = n (drug)/n (dendrimer), where n (drug) = m (encapsulated drug)/MW (drug) and n (dendrimer) = m (dendrimer)/MW (dendrimer).

**Table 8:** Zeta-potential of loaded and non-loaded metalodendrimer/dendrimer with 5-FU in filtered UPW.

Compounds	Zeta-Potential (mV)
G2.5COO(DACHPt)_16_	−10.8 ± 0.3
G2.5COO(DACHPt)_16_/5FU	0.8 ± 0.1
G2.5(COONa)_32_	−48 ± 1
G2.5(COONa)_32_/5FU	−41.1 ± 0.5

## Data Availability

The data presented in this study are available on request from the corresponding author.

## Supplementary Materials

The following are available online, Figures S1–S37, Table S1: Molecular weight of the DACHPt metallodendrimers, Schemes S1–S6.

## Sample Availability

Samples of the prepared materials are available from the authors.
