# Supplementary material for: Use of Half-Generation PAMAM Dendrimers (G0.5–G3.5) with Carboxylate End-Groups to Improve the DACHPtCl2 and 5-FU Efficacy as Anticancer Drugs
Source: Molecules. 2021 May 14;26(10):2924. doi: 10.3390/molecules26102924 (PMC8156256; doi:10.3390/molecules26102924)
Supplement: Supplementary file 1 [file molecules-26-02924-s001.zip › molecules-1191631-supplementary.pdf]

Supplementary Materials

Use of half-generation PAMAM dendrimers (G0.5-G3.5) with carboxylate end-groups to improve the $DACHPtCl_2$ and 5-FU efficacy as anticancer drugs†

Cláudia Camacho [1], Helena Tomás [1] and João Rodrigues [1,2,*]

[1] CQM-Centro de Química da Madeira, MMRG, Universidade da Madeira, Campus da Penteada, 9000-390 Funchal, Portugal;
[2] School of Materials Science and Engineering, Center for Nano Energy Materials, Northwestern Polytechnical University, Xi'an 710072, China
† Dedicated to Prof. J.-P. Majoral on his 80th Birthday.

Figure Index

Figure S1: ^{1}H-NMR spectrum of $DACHPtCl_2$ performed in D_2O. 4

Figure S2: FTIR spectra of $DACHPtCl_2$ in KBr pellet. 4

Figure S3: a) Absorption spectra of $DACHPtCl_2$ recorded at a concentration of 40μM of in ultrapure water and b) Emission (λex = 380nm) spectra of $DACHPtCl_2$ recorded at a concentration of 500μM in ultrapure water. 5

Figure S4: ^{1}H-NMR spectrum of bis-aquated DACHPt done in D_2O. 5

Figure S5: ^{13}C-NMR spectrum of bis-aquated DACHPt done in D_2O. 5

Figure S6: TOF-MS (MALDI) mass spectrum of G0.5COO$(DACHPt)_4$ metallodendrimer. 7

Figure S7: TOF-MS (ESI +) mass spectrum of G1.5COO$(DACHPt)_8$ metallodendrimer. 8

Figure S8: TOF-MS (ESI +) mass spectrum of G2.5COO$(DACHPt)_{16}$ metallodendrimer. 8

Figure S9: TOF-MS (MALDI) mass spectrum of G3.5COO$(DACHPt)_{32}$ metallodendrimer. 9

Figure S10: ^{195}Pt-NMR spectrum of G0.5COO$(DACHPt)_4$ performed in D_2O, with K_2PtCl_4 as external reference (-1631 ppm). 9

Figure S11: ^{1}H-NMR spectrum of G1.5COO$(DACHPt)_8$ performed in D_2O. 10

Figure S12: ^{13}C-NMR spectrum of G1.5COO$(DACHPt)_8$ performed in D_2O. 10

Figure S13: ^{195}Pt-NMR spectrum of G1.5COO(DACHPt)$_8$ performed in D_2O, with K_2PtCl_4 as external reference (-1631 ppm). The other signals at -1467 ppm probably corresponds to trans-[Pt(cyclopentylamine)$_2$(D_2O)$_2$](NO_3)$_2$ [1]. 11

Figure S14: ^{1}H-NMR spectrum of G2.5COO(DACHPt)$_{16}$ performed in D_2O. 11

Figure S15: ^{13}C-NMR spectrum of G2.5COO(DACHPt)$_{16}$ performed in D_2O. 12

Figure S16: ^{195}Pt-NMR spectrum of G2.5COO(DACHPt)$_{16}$ performed in D_2O, with K_2PtCl_4 as external reference (-1631 ppm). 12

Figure S17: ^{1}H-NMR spectrum of G3.5COO(DACHPt)$_{32}$ performed in D_2O. 13

Figure S18: ^{13}C-NMR spectrum of G3.5COO(DACHPt)$_{32}$ performed in D_2O. 13

Figure S19: ^{195}Pt-NMR spectrum of G3.5COO(DACHPt)$_{32}$ performed in D_2O, with K_2PtCl_4 as external reference (-1631 ppm). 14

Figure S20: FTIR spectra of different generations of anionic PAMAM dendrimers (G0.5-G3.5) in KBr pellet. 14

Figure S21: FTIR spectra of DACHPt-metallodendrimers in KBr pellet. 15

Figure S22: UV-Vis spectra of anionic PAMAM dendrimers at a concentration of 500 μM in ultrapure water. 15

Figure S23: Emission (λex= 380nm) of anionic PAMAM dendrimers at a concentration of 500μM in ultrapure water. 16

Figure S24: Representative UV-visible spectra of DACHPtCl$_2$ with increasing concentration of CT-DNA (0, 6.25, 12.5, 18.75, 25, 31.25, 37.5, 43.75 and 50 μM) in 5 mM Tris-HCl/50 mM NaCl at pH 7.4. The inset corresponds to the plot of A0/(A-A0) versus 1/[DNA], which is used to determine the binding constant. The arrow indicates the direction of increasing the concentration of DNA. 16

Figure S25: Representative UV-visible spectra of oxaliplatin with increasing concentration of CT-DNA (0, 6.25, 12.5, 18.75, 25, 31.25, 37.5, 43.75 and 50 μM) in 5 mM Tris-HCl/50 mM NaCl at pH 7.4. The inset corresponds to the plot of A0/(A-A0) versus 1/[DNA], which is used to determine the binding constant. The arrow indicates the direction of increasing the concentration of DNA. 17

Figure S26: FTIR spectra of G2.5COO(DACHPt)$_{16}$/5-FU in KBr pellet. 17

Figure S27: FTIR spectra of G2.5(COONa)$_{32}$/5-FU in KBr pellet. 18

Figure S28: ^{1}H-NMR of G2.5COO(DACHPt)$_{16}$, G2.5COO(DACHPt)$_{16}$/5-FU and 5-FU in D_2O. 18

Figure S29: ^{13}C-NMR of G2.5COO(DACHPt)$_{16}$, G2.5COO(DACHPt)$_{16}$/5-FU and 5-FU in D_2O. 19

Figure S30: ^{19}F-NMR of G2.5COO(DACHPt)$_{16}$/5-FU and 5-FU in D_2O. 20

Figure S31: ^{1}H-NMR of G2.5(COONa)$_{32}$, G2.5(COONa)$_{32}$/5-FU and 5-FU in D_2O............................... 21

Figure S32: ^{13}C-NMR of G2.5(COONa)$_{32}$, G2.5(COONa)$_{32}$/5-FU and 5-FU in D_2O............................. 22

Figure S33: ^{19}F-NMR of G2.5(COONa)$_{32}$/5-FU and 5-FU in D_2O.. 23

Figure S34: Standard curve of Hg using several dilutions: 0.2; 0.37; 0.54; 0.71; 0.88; 1.05; 1.22 and 1.39mg/mL. The absorbance was measured at 550nm.. 25

Figure S35: Standard curve of 5-Fluorouracil in ultrapure water using different concentrations: 0.25, 0.5, 0.75, 1, 2.5, 5, 7.5, 10, 15, 20 and 25 μg/mL. The absorbance was measured at 266nm. 26

Figure S36: Standard curve of 5-Fluorouracil in PBS 5 using different concentrations: 0.25, 0.5, 0.75, 1, 2.5, 5, 7.5, 10, 15, 20 and 25 μg/mL. The absorbance was measured at 266nm. 26

Figure S37: Standard curve of 5-Fluorouracil in PBS 7.4 using different concentrations: 0.25, 0.5, 0.75, 1, 2.5, 5, 7.5, 10, 15, 20 and 25 μg/mL. The absorbance was measured at 266nm. 27

Table Index

Table S1: Molecular weight of the DACHPt metallodendrimers. 6

Scheme Index

Scheme S1: Synthesis of DACHPtCl$_2$ compound. 23

Scheme S2: Synthesis of DACHPt(H_2O)$_2$ compound. 24

Scheme S3: Representation of the synthesis of anionic PAMAM dendrimer G0.5 with DACHPt(H_2O)$_2$ complex, G0.5COO(DACHPt)$_4$. 24

Scheme S4: Representation of the synthesis of anionic PAMAM dendrimer G1.5 with DACHPt(H_2O)$_2$ complex, G1.5COO(DACHPt)$_8$. 24

Scheme S5: Representation of the synthesis of anionic PAMAM dendrimer G2.5 with DACHPt(H_2O)$_2$ complex, G2.5COO(DACHPt)$_{16}$. 25

Scheme S6: Representation of the synthesis of anionic PAMAM dendrimer G2.5 with DACHPt(H_2O)$_2$ complex, G3.5COO(DACHPt)$_{32}$. 25

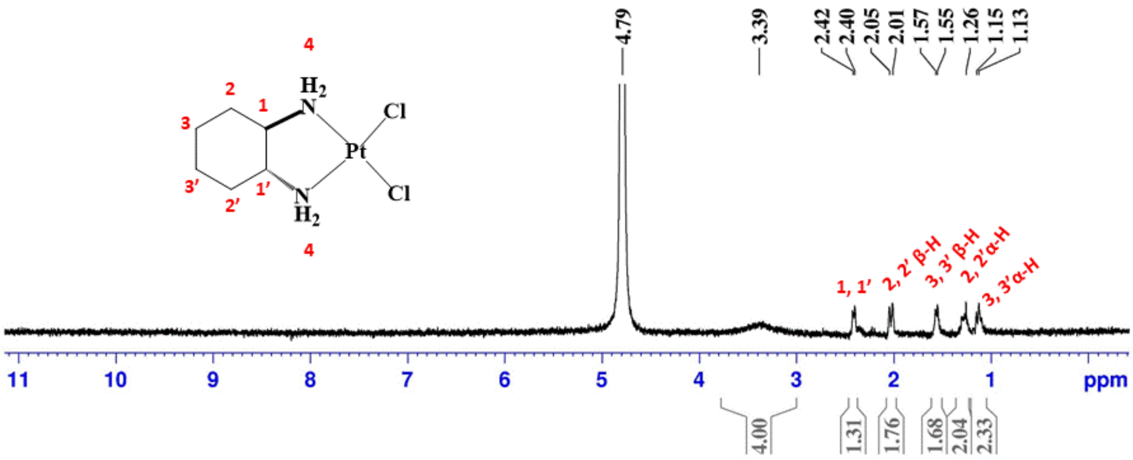

Figure S1: ^{1}H-NMR spectrum of $DACHPtCl_2$ performed in D_2O.

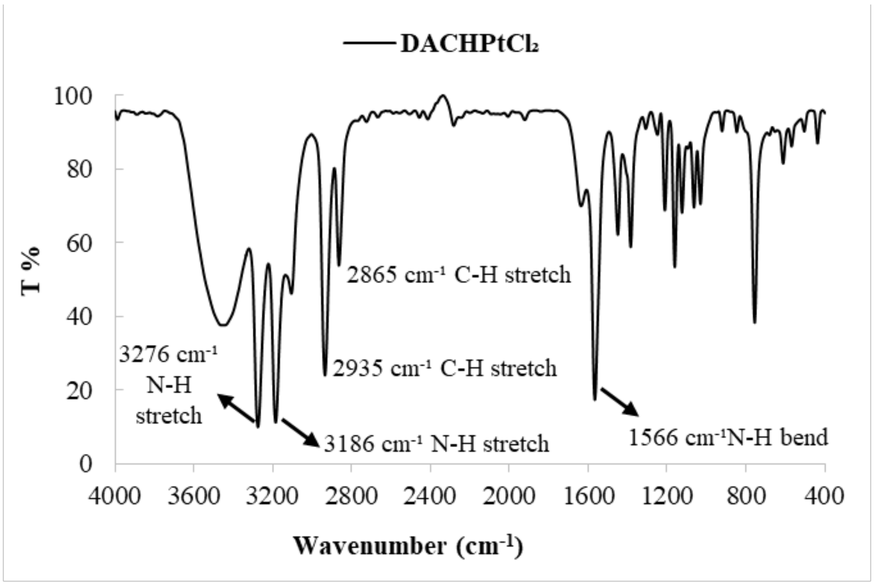

Figure S2: FTIR spectra of $DACHPtCl_2$ in KBr pellet.

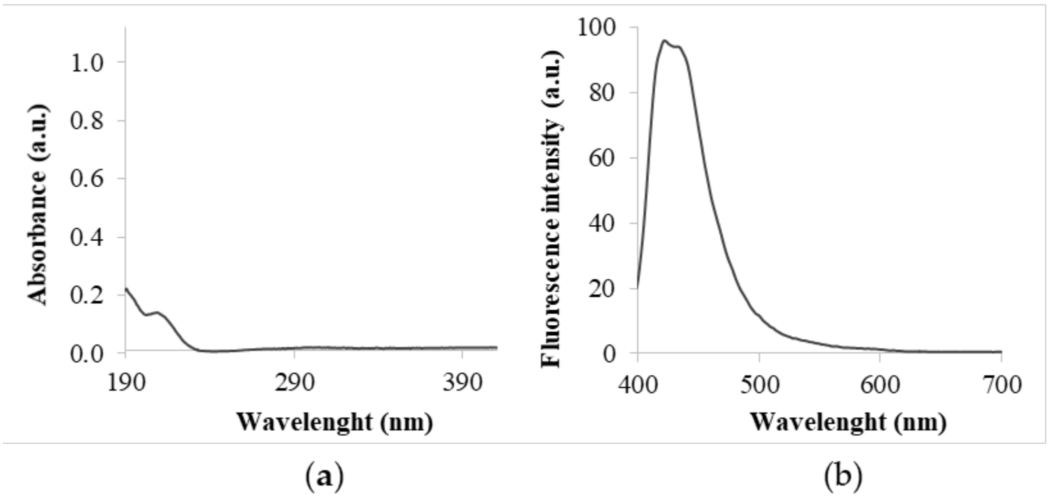

Figure S3: a) Absorption spectra of $DACHPtCl_2$ recorded at a concentration of 40µM of in ultrapure water and b) Emission (λex = 380nm) spectra of $DACHPtCl_2$ recorded at a concentration of 500µM in ultrapure water.

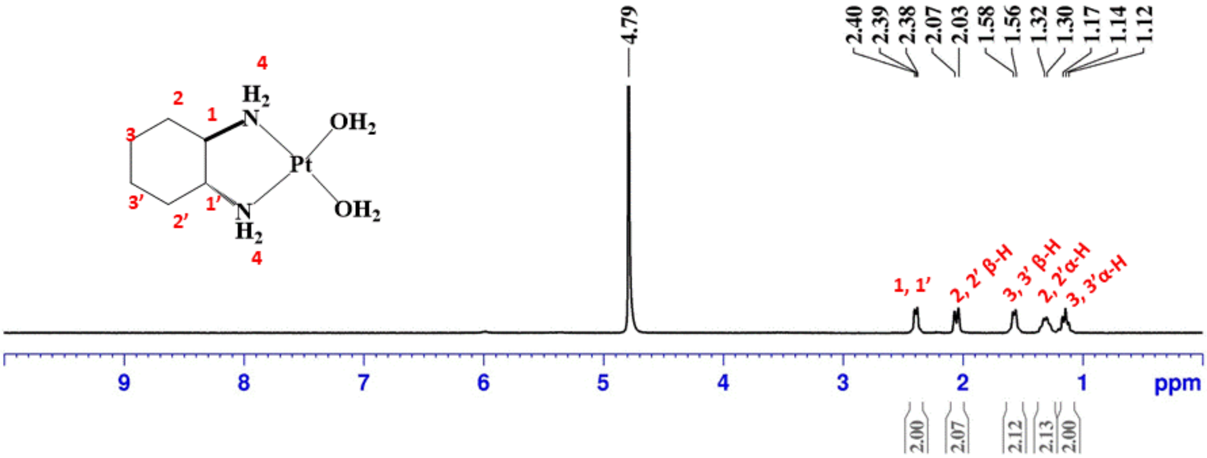

Figure S4: ^{1}H-NMR spectrum of bis-aquated DACHPt done in D_2O.

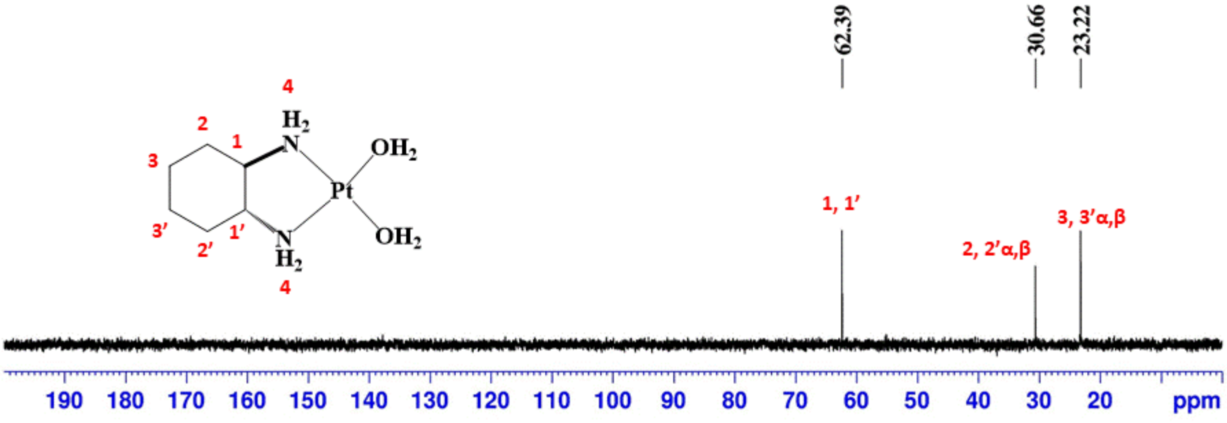

Figure S5: ^{13}C-NMR spectrum of bis-aquated DACHPt done in D_2O.

Table S1: Molecular weight of the DACHPt metallodendrimers.

	$G0.5COO(DACHPt)_4$	$G1.5COO(DACHPt)_8$	$G2.5COO(DACHPt)_{16}$	$G3.5COO(DACHPt)_{32}$
**Molecular weight**	2322.24	5040.96	10478.42	21353.33
**m/z calculated**	2014.8	1008.58	1558.7	388.9
**m/z found**	2014.7<br>$[M+H]^+$<br><br>$C_{64}H_{117}N_{16}O_{20}Pt_3^+$	1008.98<br>$[M+5H]^{5+}$<br><br>$C_{158}H_{293}N_{42}O_{44}Pt_8{}^{5+}$	1556.7<br>$[M+H^++8MeOH]^+$<br><br>$C_{342}H_{641}N_{90}O_{100}Pt_{16}^+$	388.2<br>[M]<br><br>$C_{632}H_{1141}N_{168}O_{188}Pt_{23}$

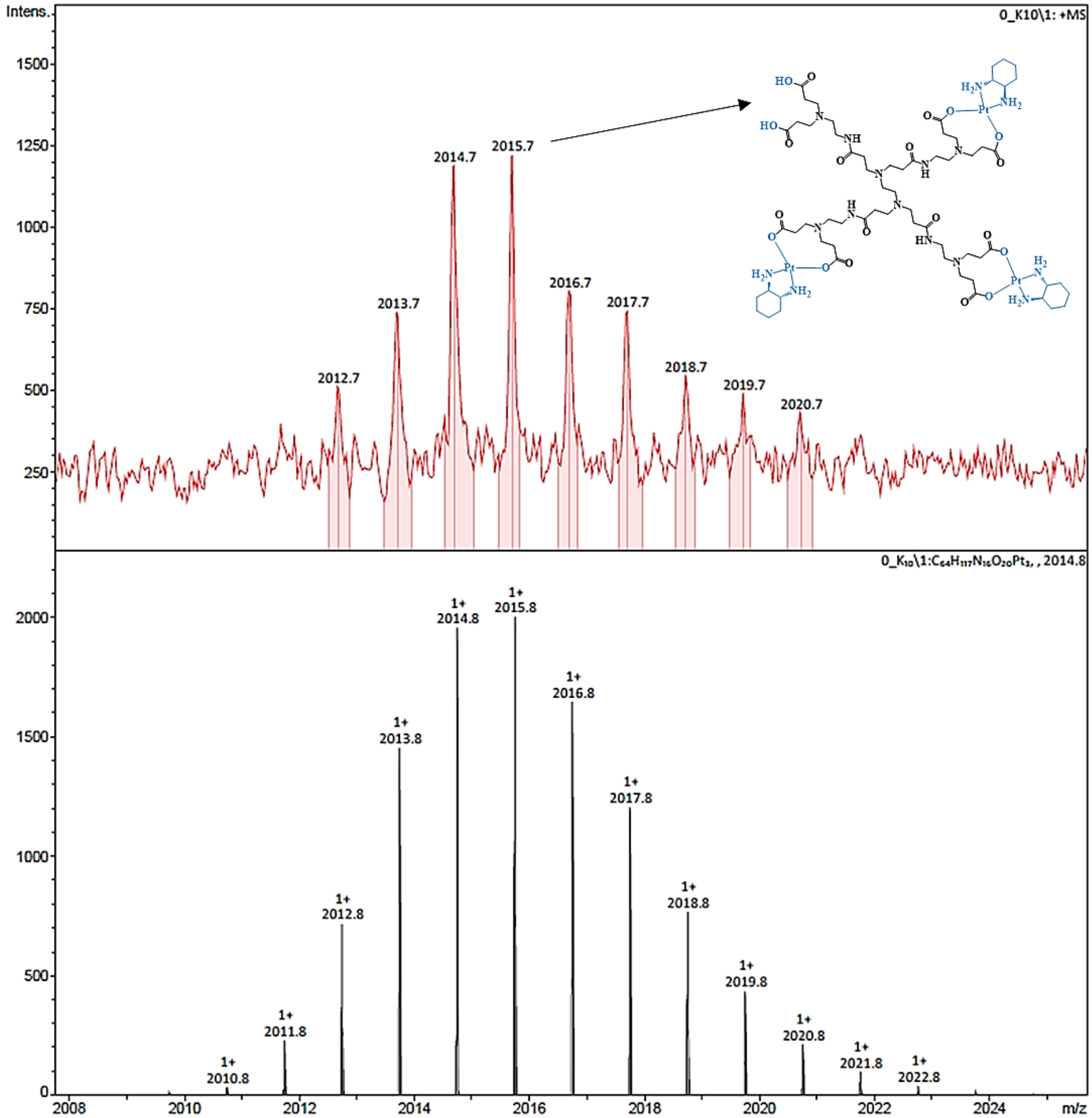

Figure S6: TOF-MS (MALDI) mass spectrum of G0.5COO(DACHPt)4 metallodendrimer.

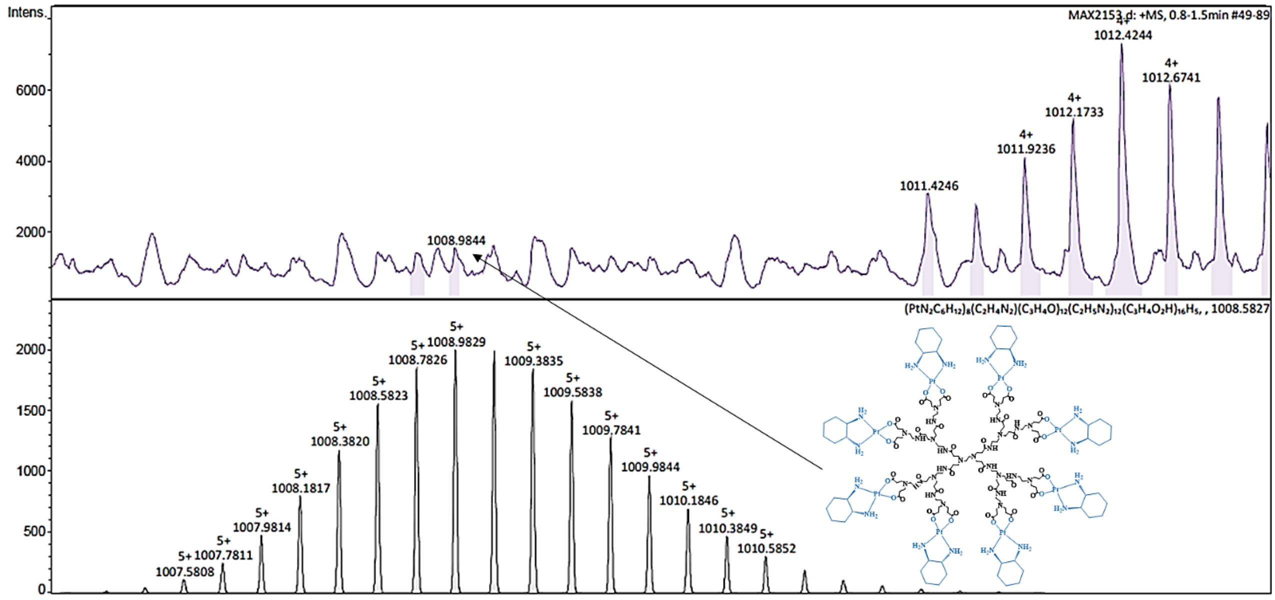

Figure S7: TOF-MS (ESI +) mass spectrum of G1.5COO(DACHPt)8 metallodendrimer.

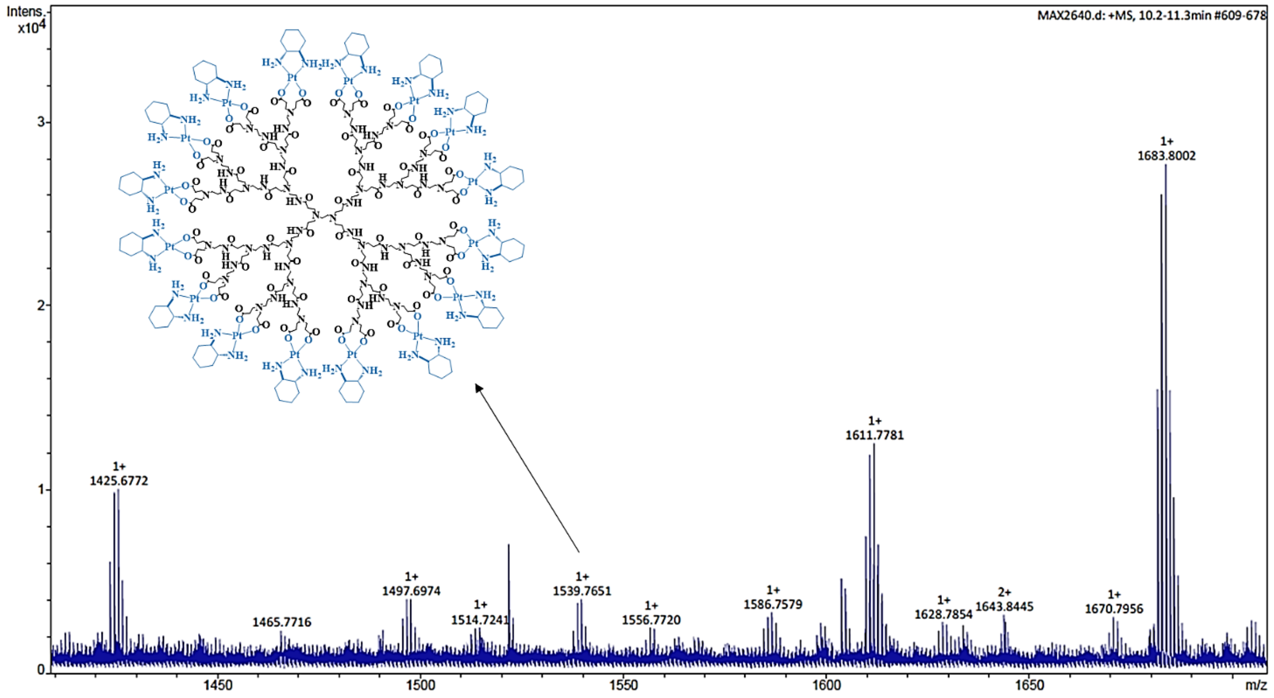

Figure S8: TOF-MS (ESI +) mass spectrum of G2.5COO(DACHPt)16 metallodendrimer.

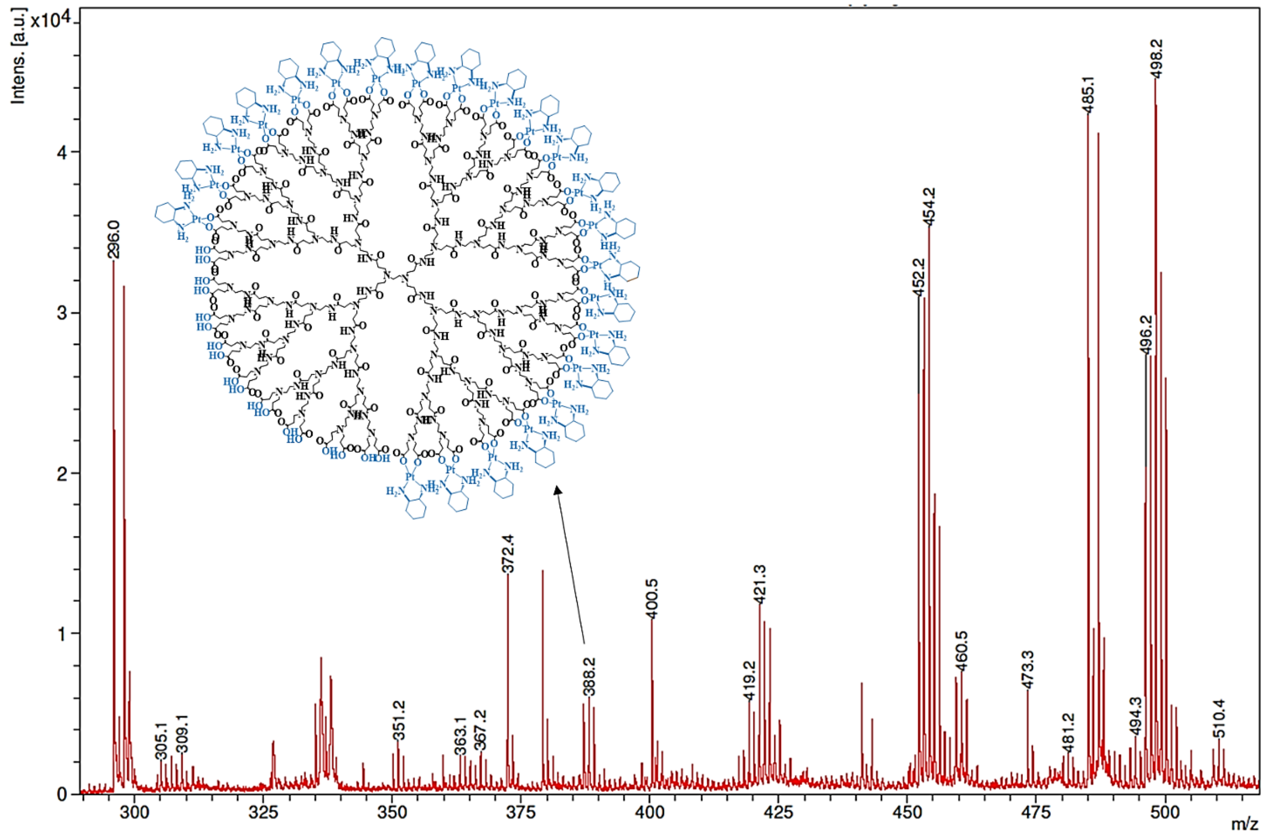

Figure S9: TOF-MS (MALDI) mass spectrum of G3.5COO(DACHPt)$_{32}$ metallodendrimer.

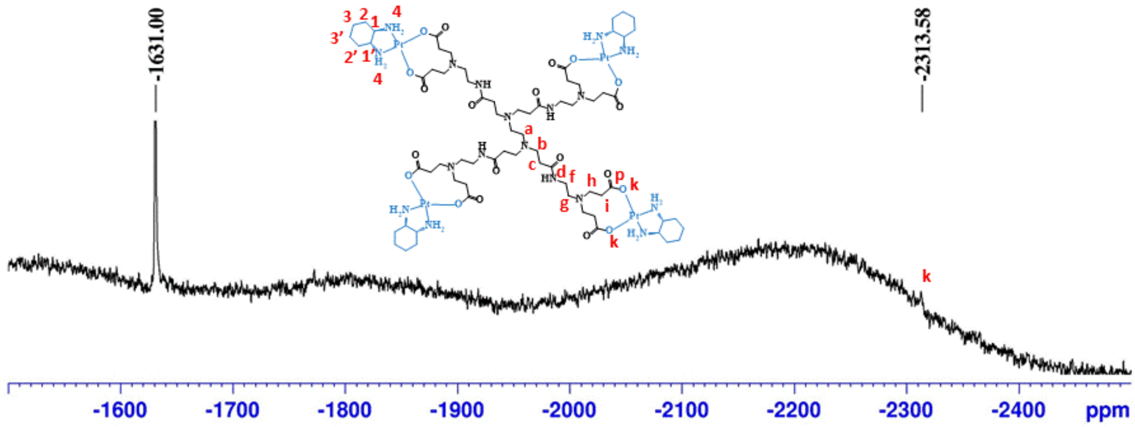

Figure S10: ^{195}Pt-NMR spectrum of G0.5COO(DACHPt)$_4$ performed in D_2O, with K_2PtCl_4 as external reference (-1631 ppm).

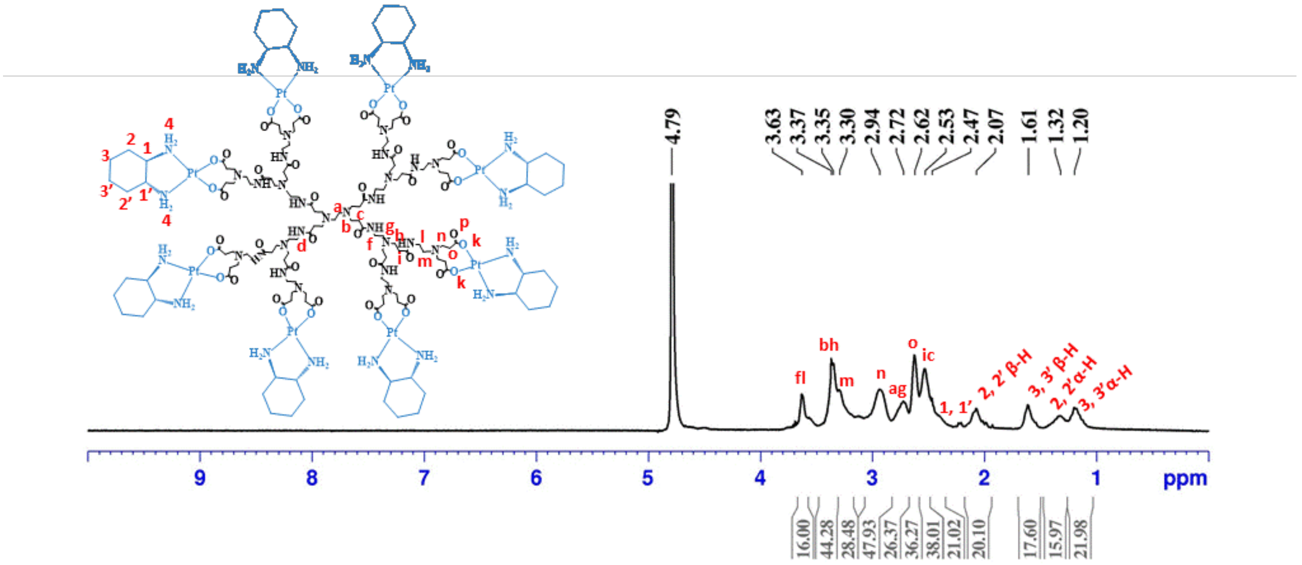

Figure S11: ^{1}H-NMR spectrum of G1.5COO(DACHPt)$_8$ performed in D_2O.

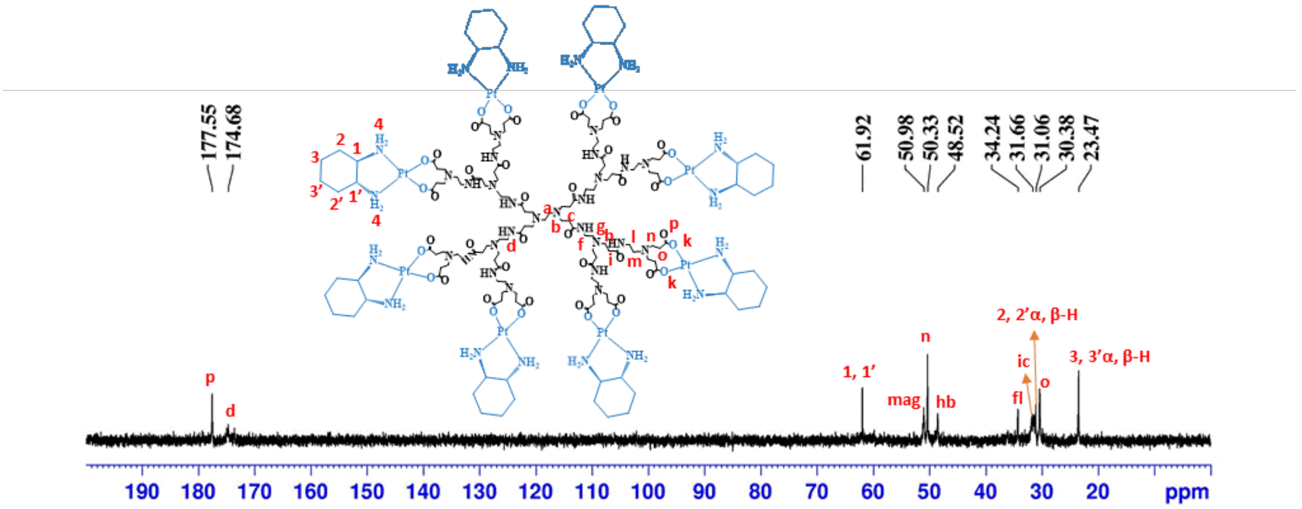

Figure S12: ^{13}C-NMR spectrum of G1.5COO(DACHPt)$_8$ performed in D_2O.

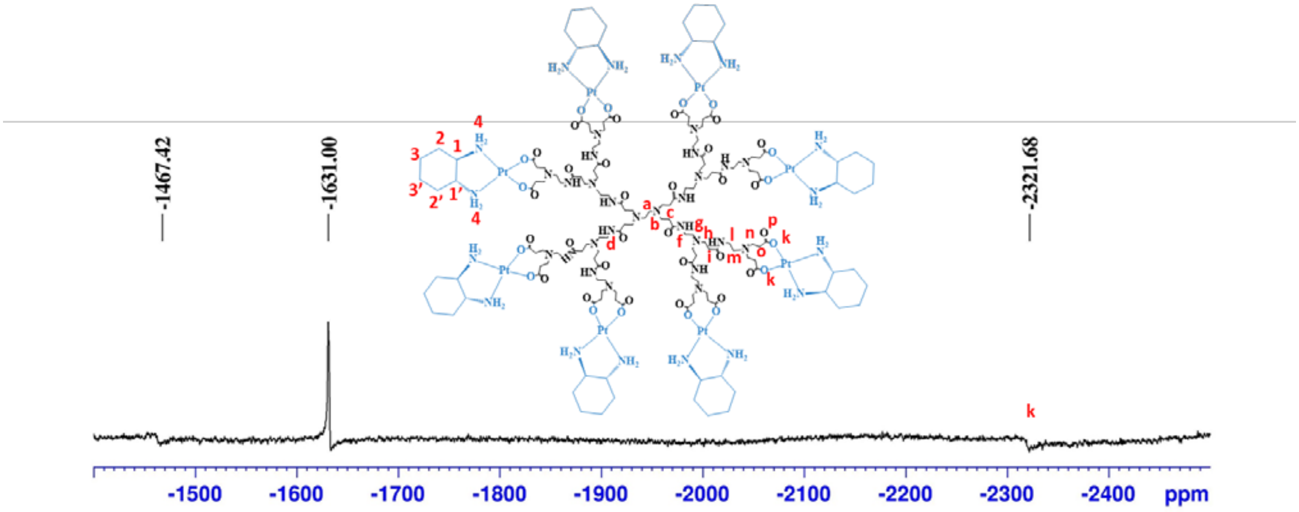

Figure S13: ^{195}Pt-NMR spectrum of G1.5COO(DACHPt)$_8$ performed in D_2O, with K_2PtCl_4 as external reference (-1631 ppm). The other signals at -1467 ppm probably corresponds to trans-[Pt(cyclopentylamine)$_2$(D_2O)$_2$](NO_3)$_2$ [1].

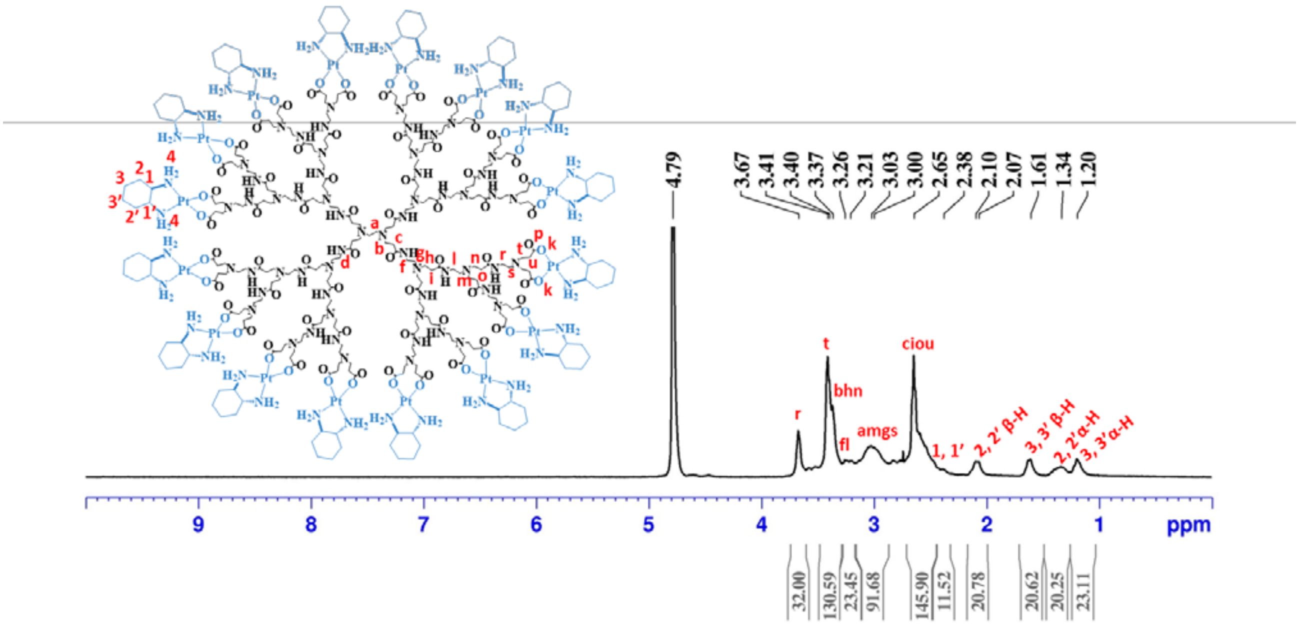

Figure S14: ^{1}H-NMR spectrum of G2.5COO(DACHPt)$_{16}$ performed in D_2O.

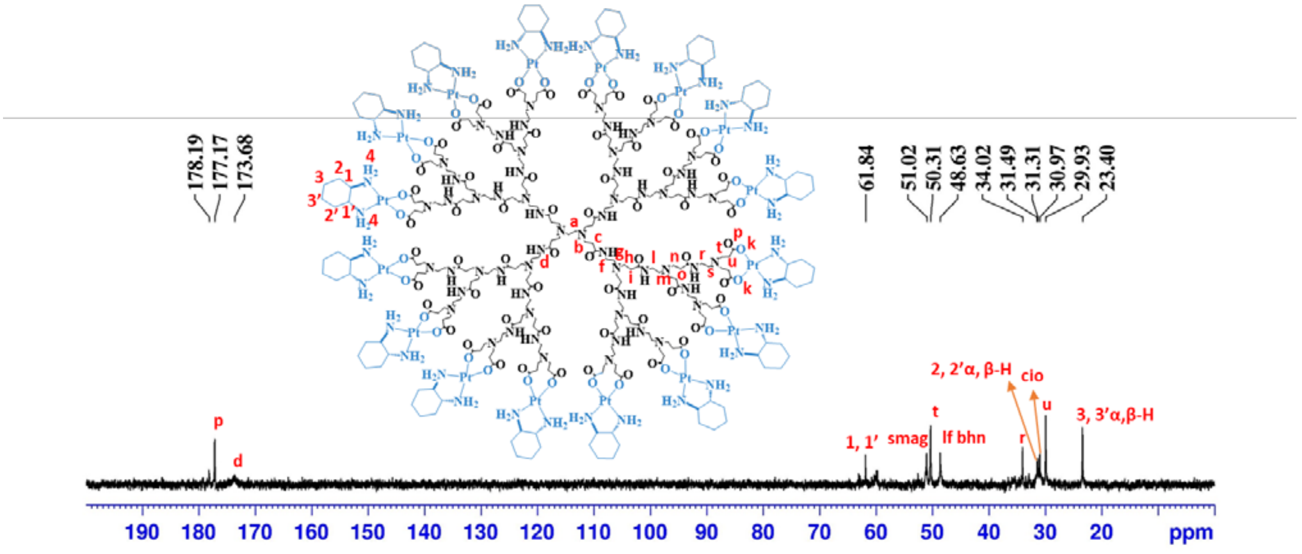

Figure S15: ^{13}C-NMR spectrum of G2.5COO(DACHPt)$_{16}$ performed in D_2O.

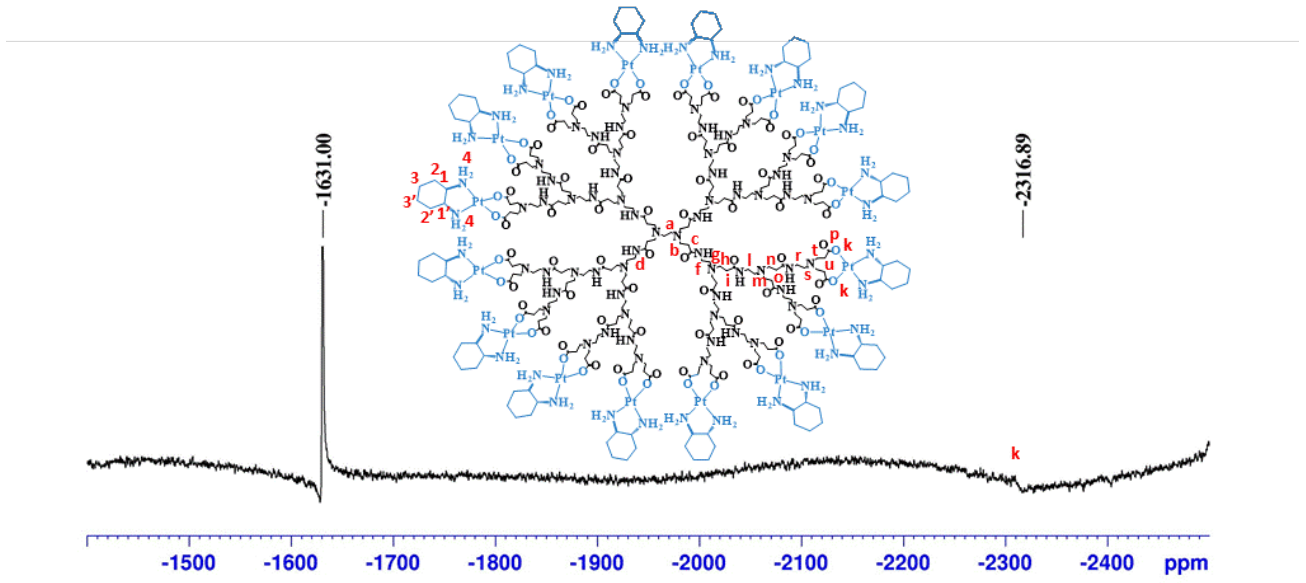

Figure S16: ^{195}Pt-NMR spectrum of G2.5COO(DACHPt)$_{16}$ performed in D_2O, with K_2PtCl_4 as external reference (-1631 ppm).

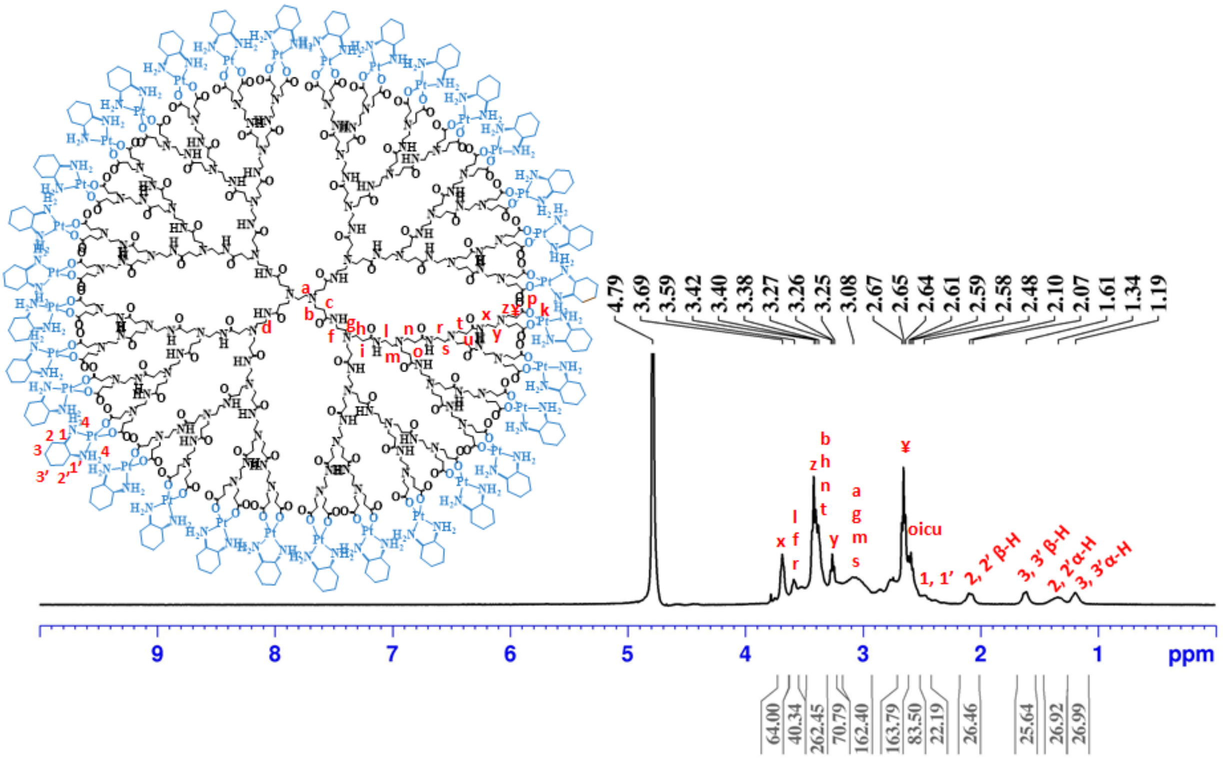

Figure S17: ^{1}H-NMR spectrum of G3.5COO(DACHPt)$_{32}$ performed in D_2O.

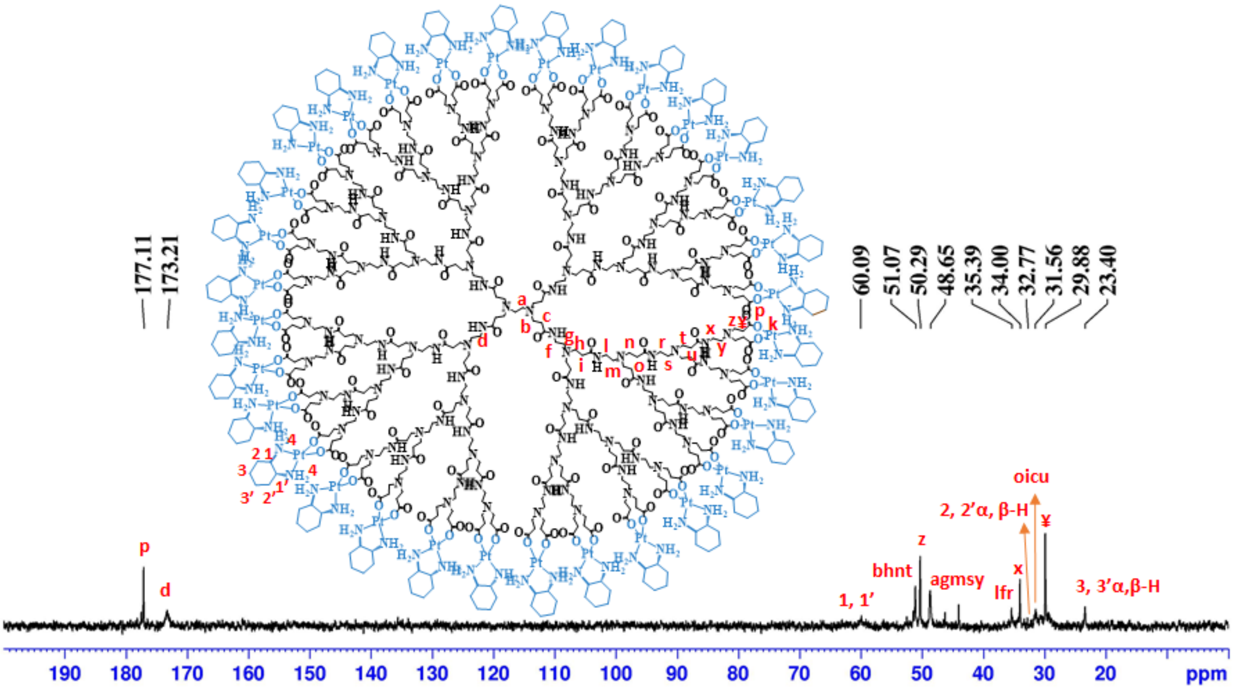

Figure S18: ^{13}C-NMR spectrum of G3.5COO(DACHPt)$_{32}$ performed in D_2O.

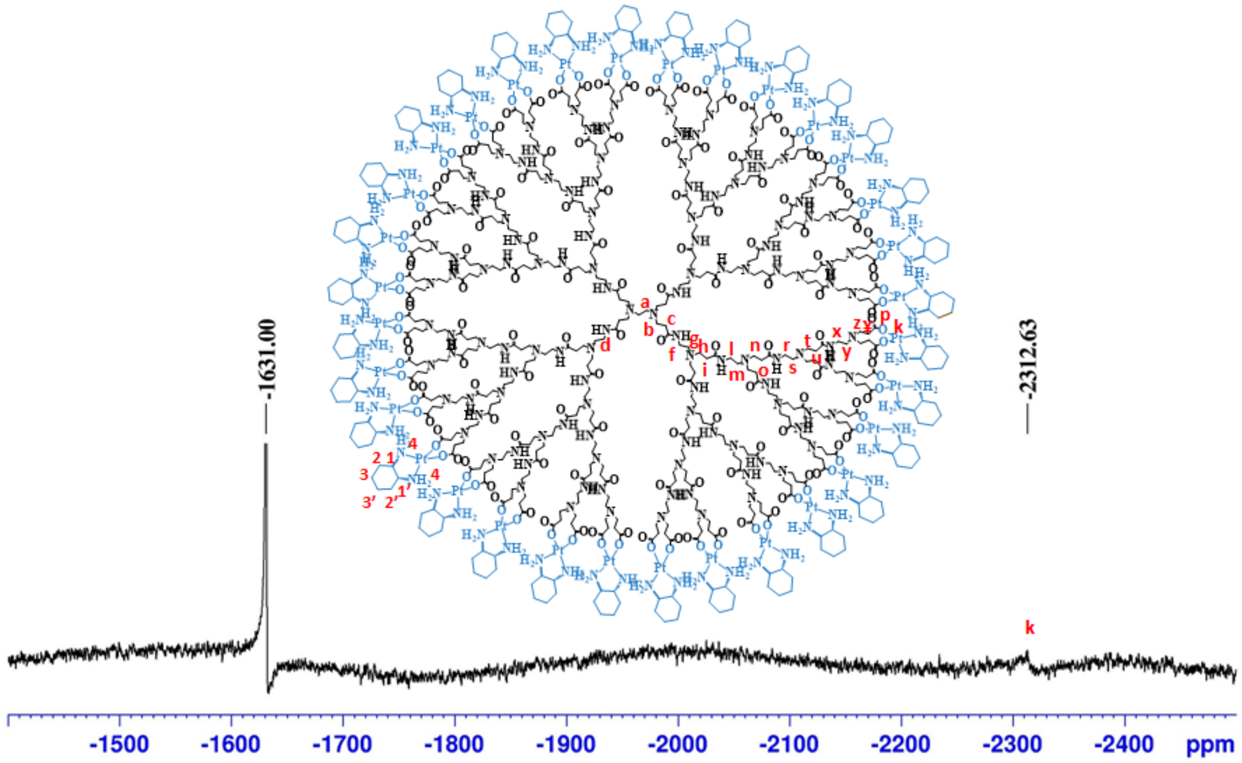

Figure S19: ^{195}Pt-NMR spectrum of G3.5COO(DACHPt)$_{32}$ performed in D_2O, with K_2PtCl_4 as external reference (-1631 ppm).

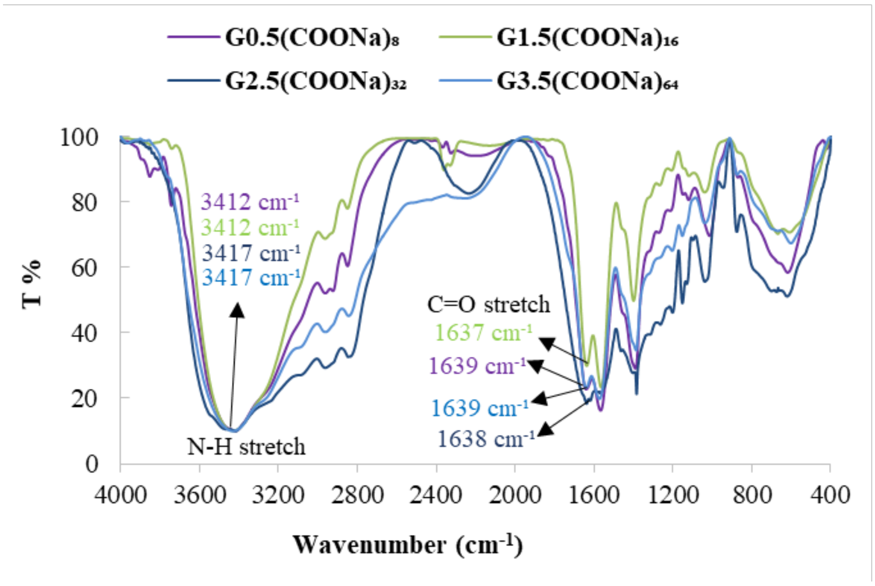

Figure S20: FTIR spectra of different generations of anionic PAMAM dendrimers (G0.5-G3.5) in KBr pellet.

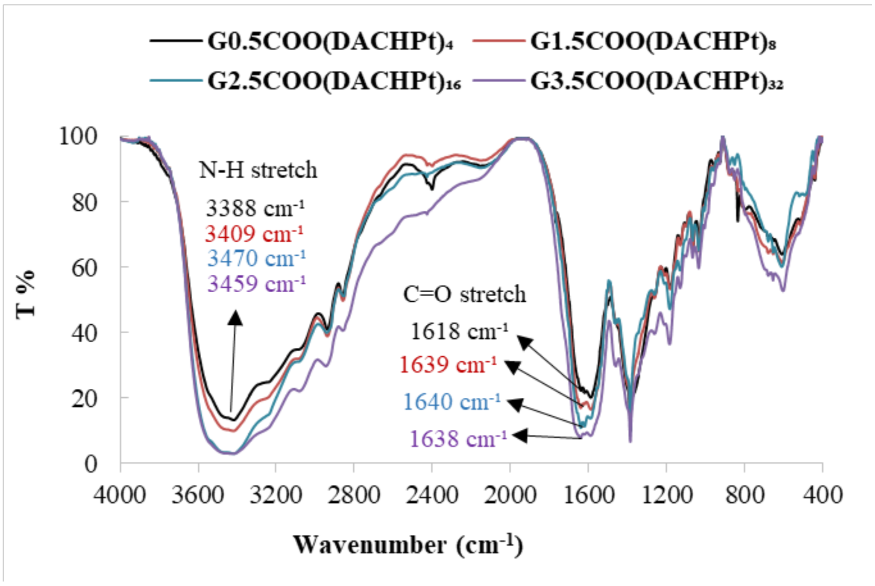

Figure S21: FTIR spectra of DACHPt metallodendrimers in KBr pellet.

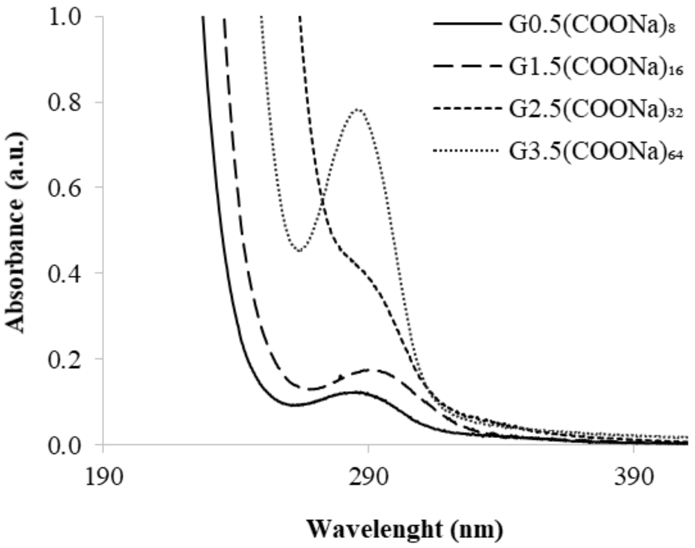

Figure S22: UV-Vis spectra of anionic PAMAM dendrimers at a concentration of 500 µM in ultrapure water.

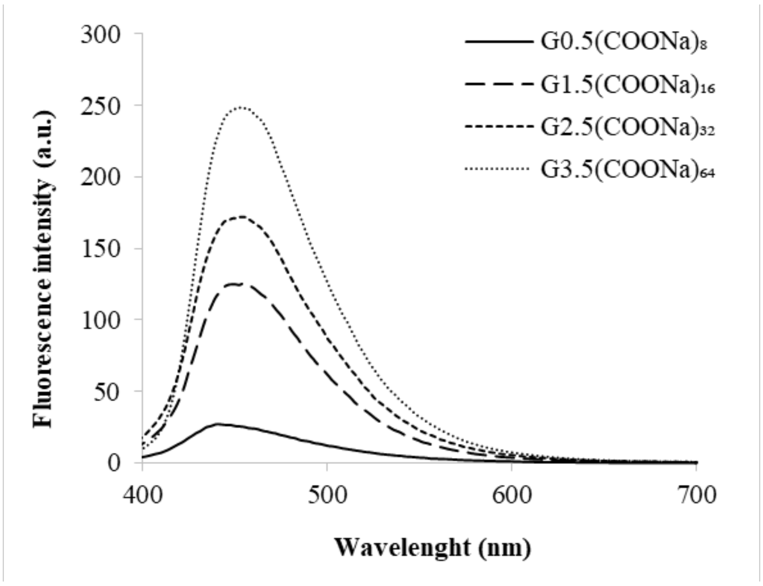

Figure S23: Emission (λex= 380nm) of anionic PAMAM dendrimers at a concentration of 500μM in ultrapure water.

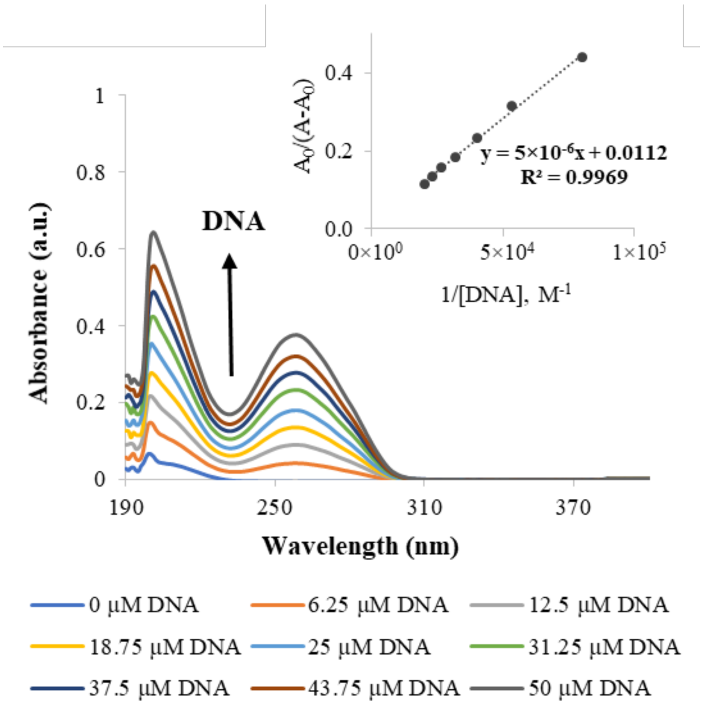

Figure S24: Representative UV-visible spectra of $DACHPtCl_2$ with increasing concentration of CT-DNA (0, 6.25, 12.5, 18.75, 25, 31.25, 37.5, 43.75 and 50 μM) in 5 mM Tris-HCl/50 mM NaCl at pH 7.4. The inset corresponds to the plot of A0/(A-A0) versus 1/[DNA], which is used to determine the binding constant. The arrow indicates the direction of increasing the concentration of DNA.

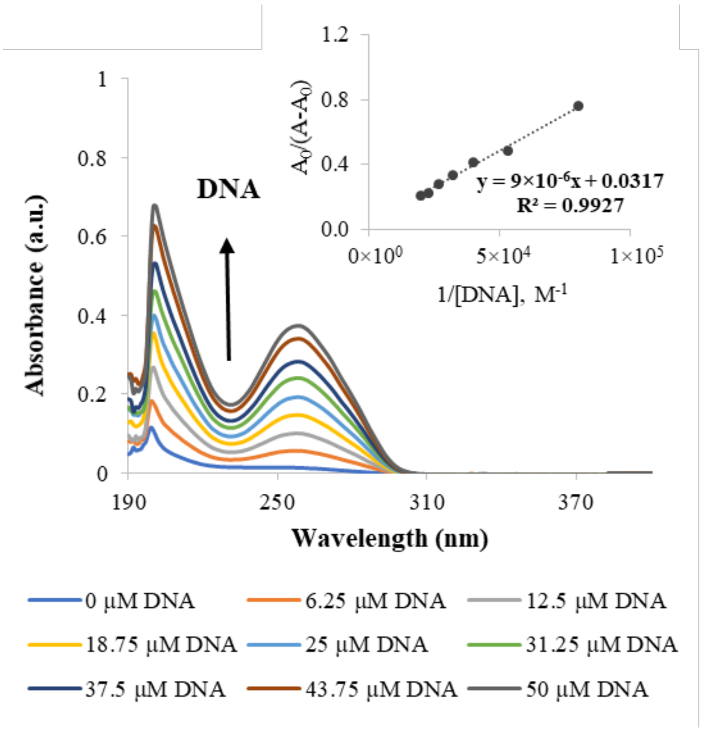

Figure S25: Representative UV-visible spectra of oxaliplatin with increasing concentration of CT-DNA (0, 6.25, 12.5, 18.75, 25, 31.25, 37.5, 43.75 and 50 µM) in 5 mM Tris-HCl/50 mM NaCl at pH 7.4. The inset corresponds to the plot of A0/(A-A0) versus 1/[DNA], which is used to determine the binding constant. The arrow indicates the direction of increasing the concentration of DNA.

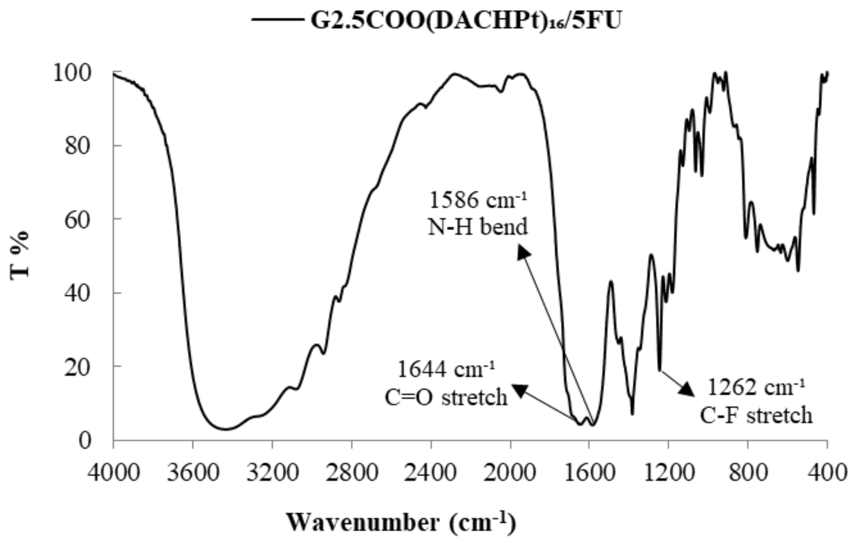

Figure S26: FTIR spectra of $G2.5COO(DACHPt)_{16}$/5-FU in KBr pellet.

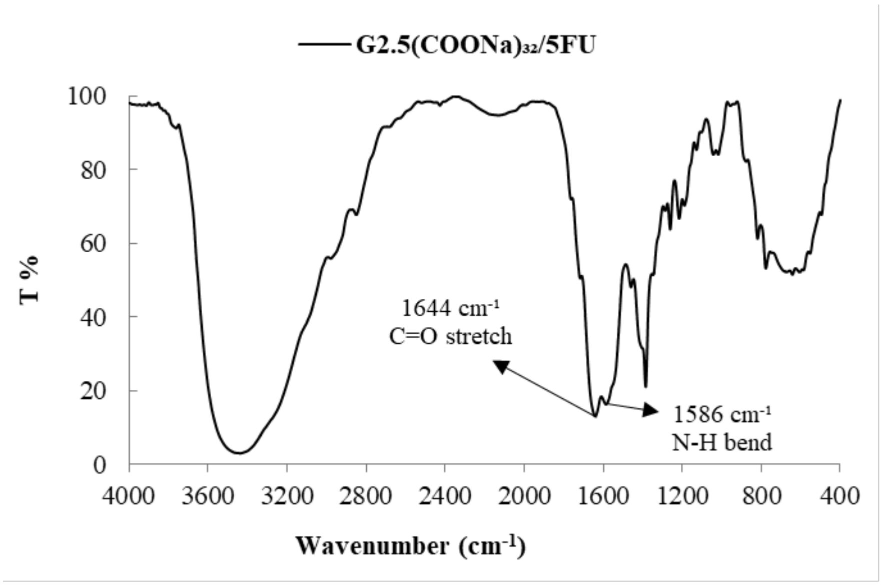

Figure S27: FTIR spectra of G2.5(COONa)32/5-FU in KBr pellet.

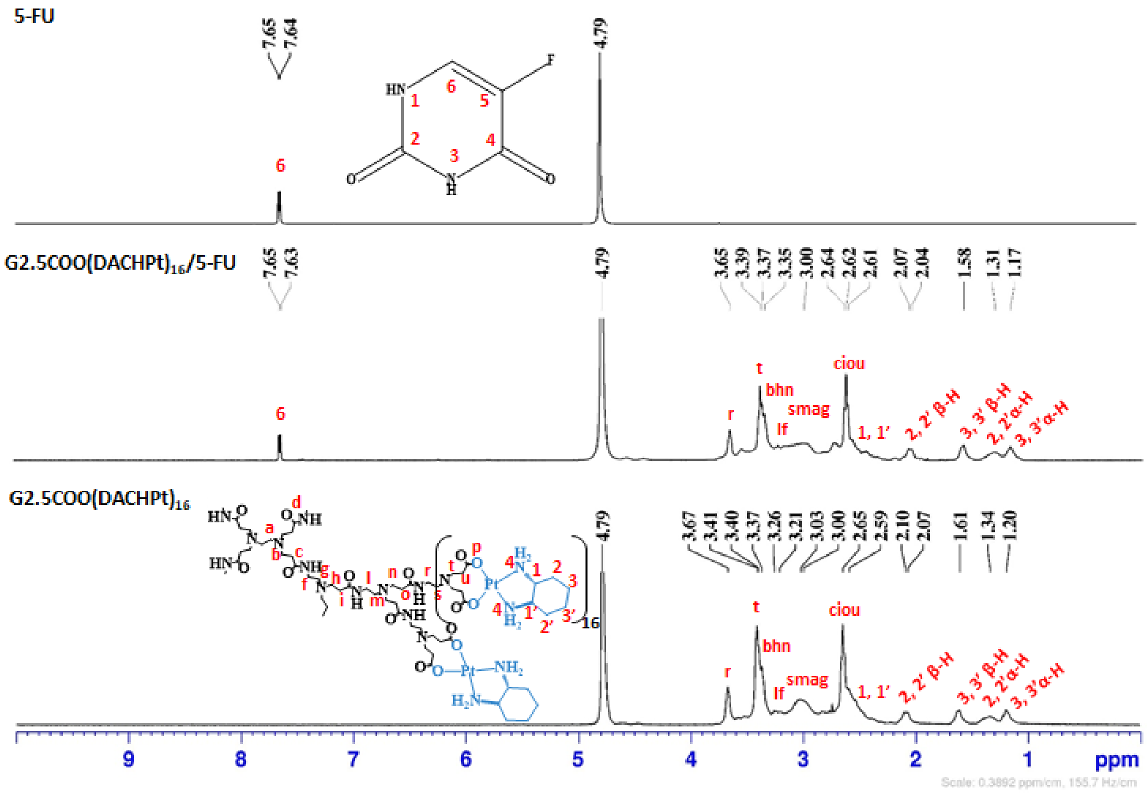

Figure S28: ^{1}H-NMR of G2.5COO(DACHPt)$_{16}$, G2.5COO(DACHPt)$_{16}$/5-FU and 5-FU in D_2O.

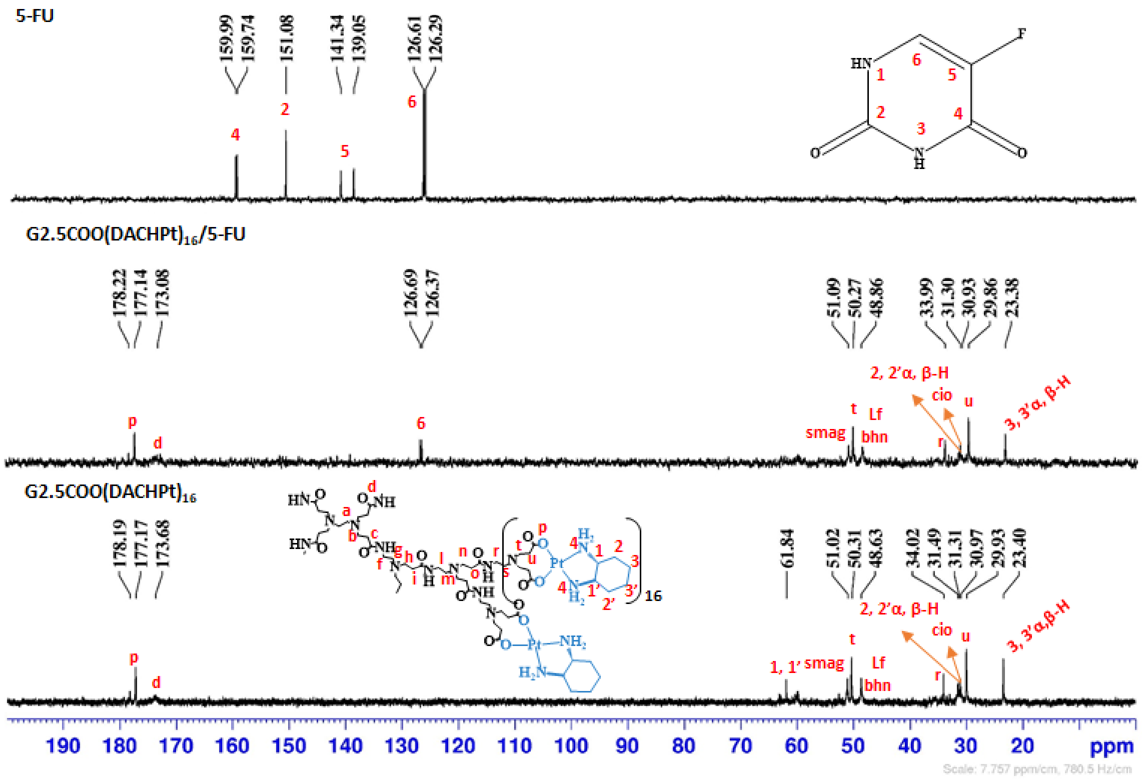

Figure S29: ^{13}C-NMR of G2.5COO(DACHPt)$_{16}$, G2.5COO(DACHPt)$_{16}$/5-FU and 5-FU in D_2O.

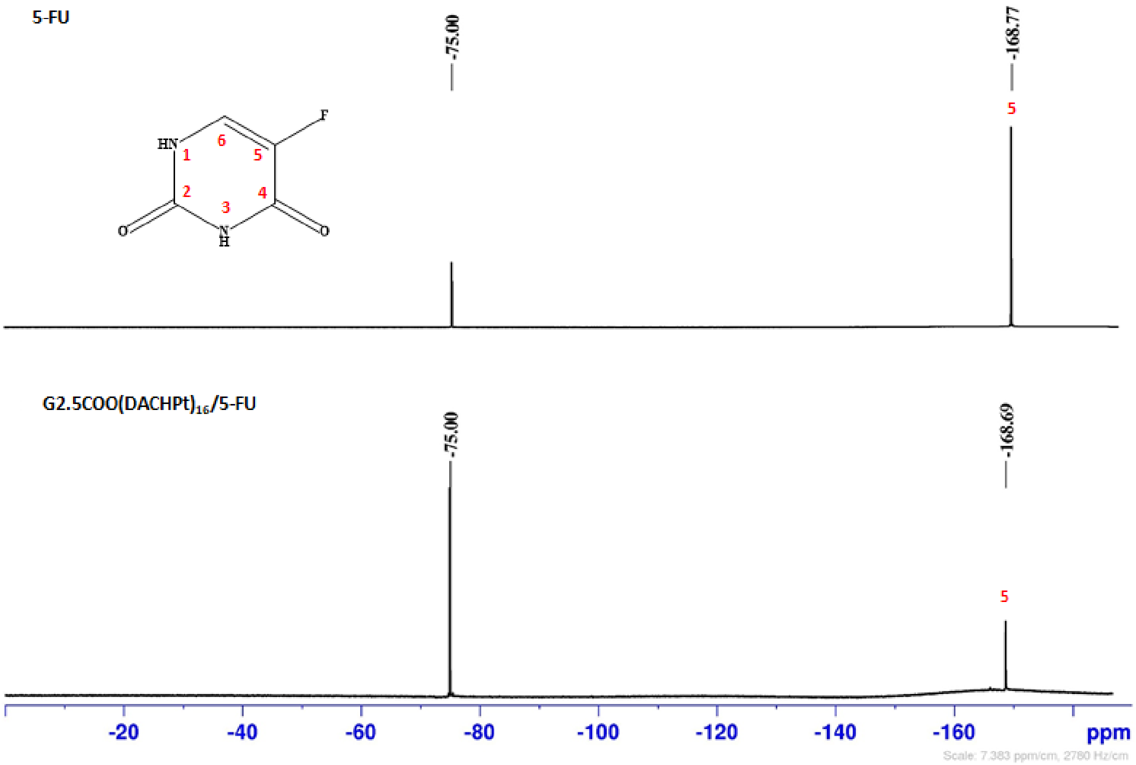

Figure S30: ^{19}F-NMR of G2.5COO(DACHPt)$_{16}$/5-FU and 5-FU in D_2O.

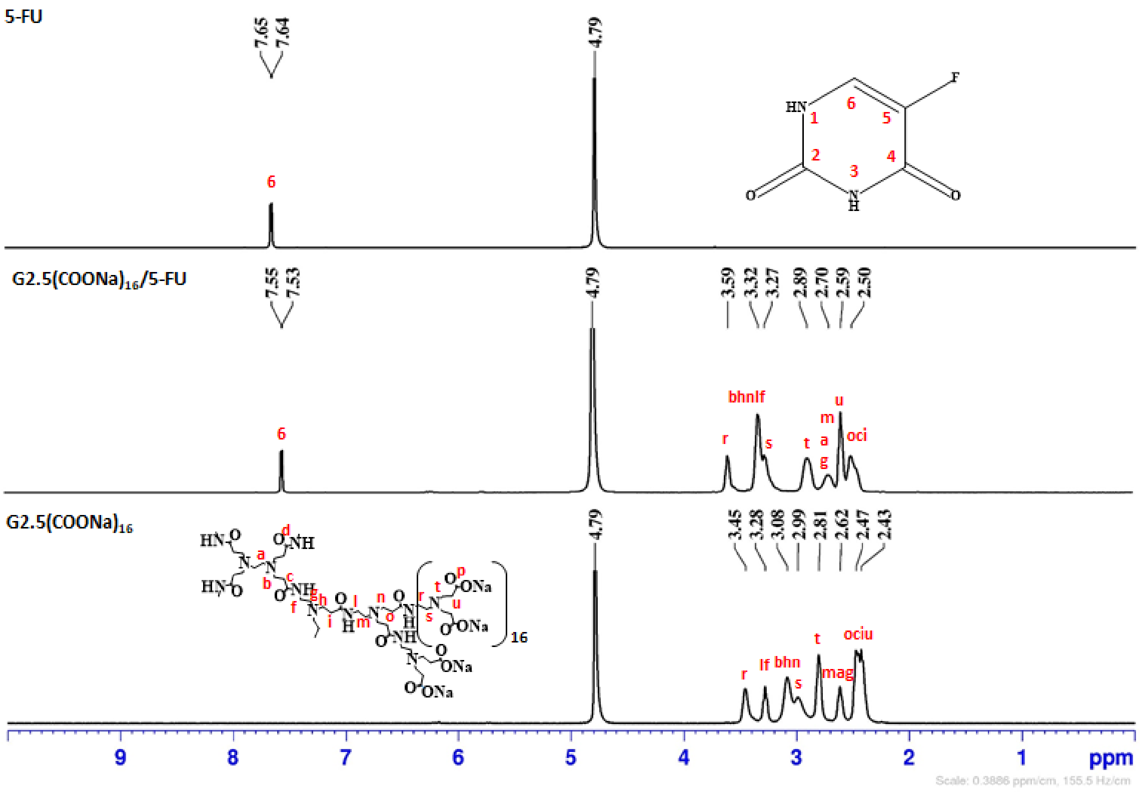

Figure S31: ^{1}H-NMR of G2.5(COONa)$_{32}$, G2.5(COONa)$_{32}$/5-FU and 5-FU in D_2O.

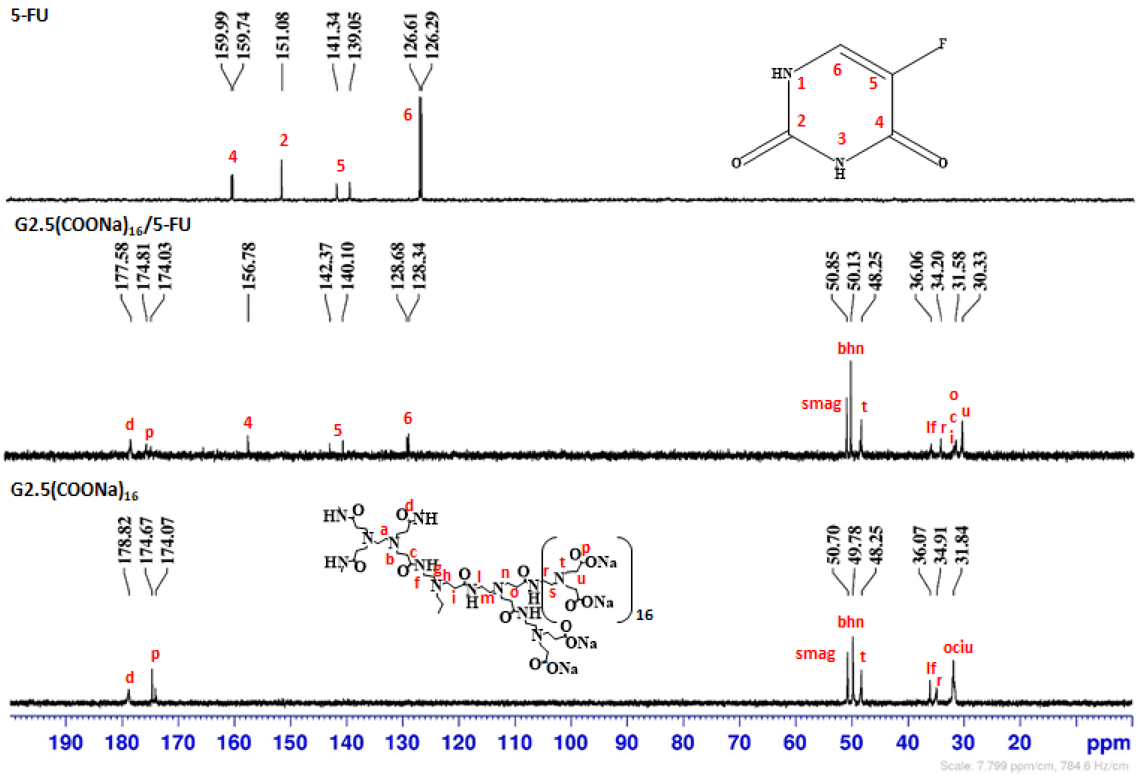

Figure S32: ^{13}C-NMR of G2.5(COONa)$_{32}$, G2.5(COONa)$_{32}$/5-FU and 5-FU in D_2O.

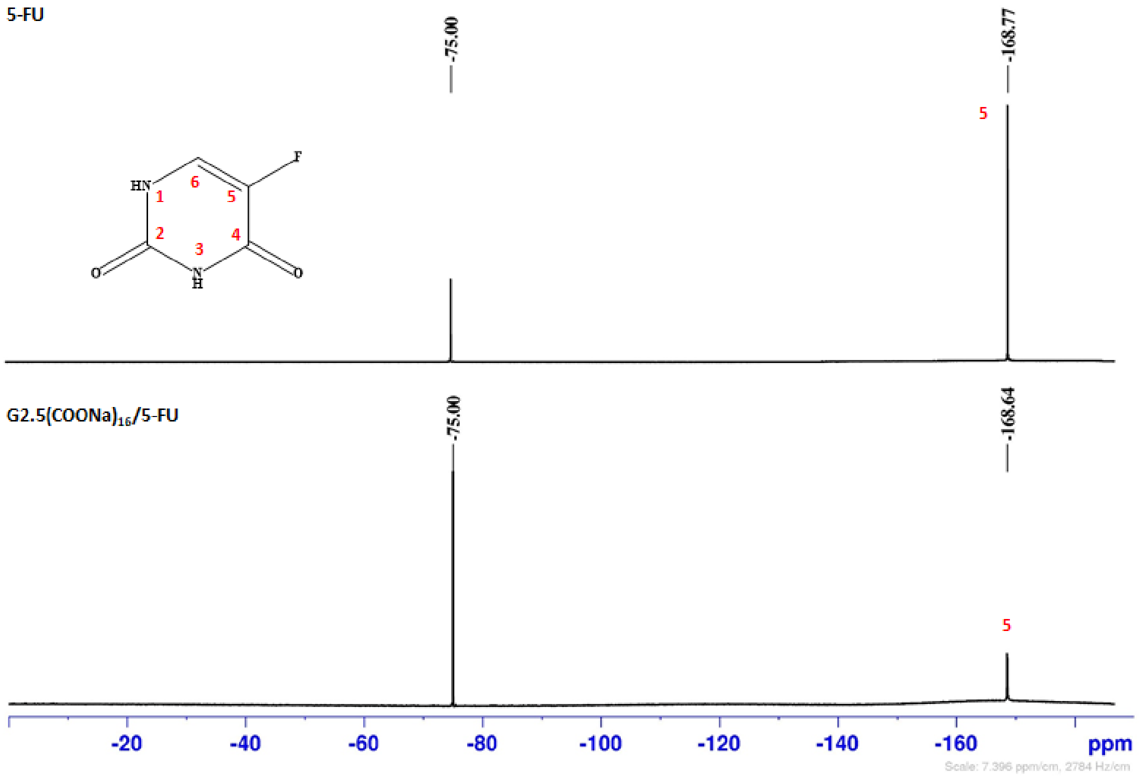

Figure S33: ^{19}F-NMR of G2.5$(COONa)_{32}$/5-FU and 5-FU in D_2O.

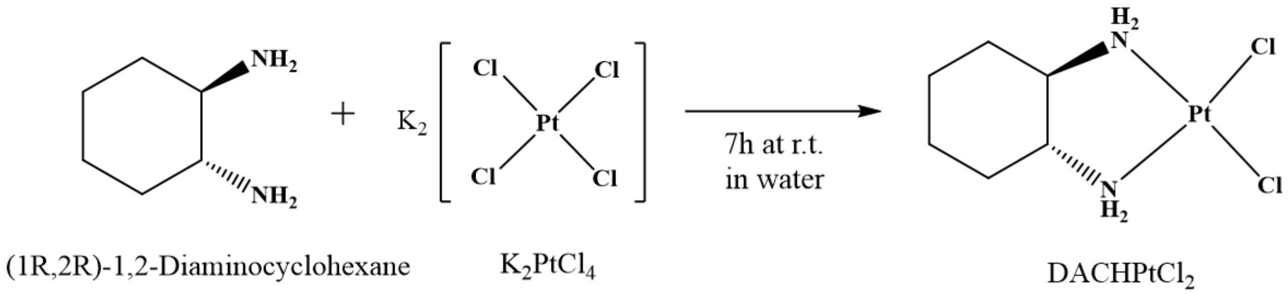

Scheme S1: Synthesis of $DACHPtCl_2$ compound.

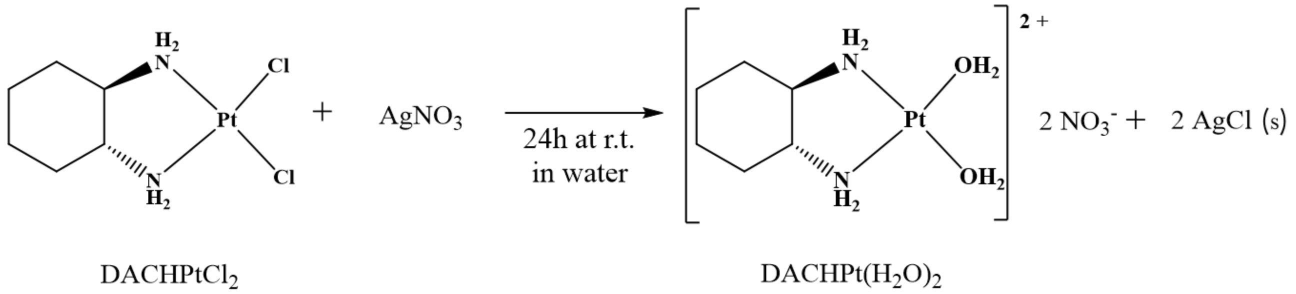

Scheme S2: Synthesis of $DACHPt(H_2O)_2$ compound.

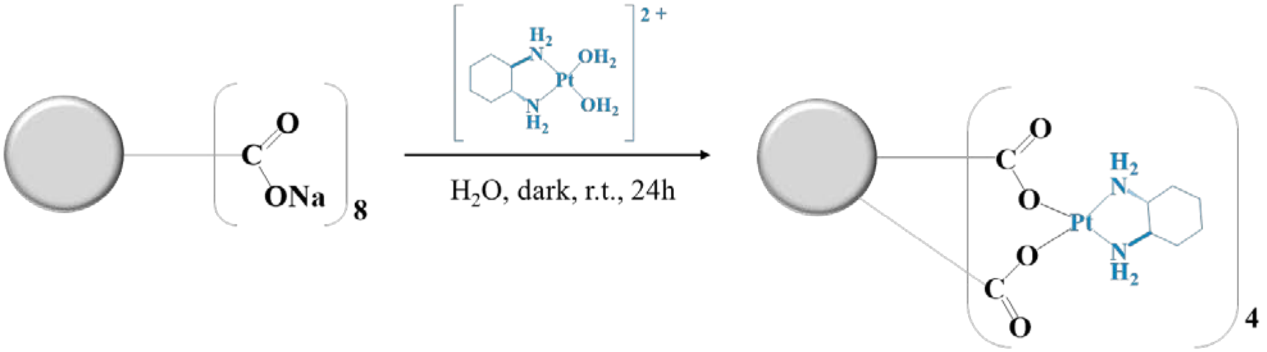

Scheme S3: Representation of the synthesis of anionic PAMAM dendrimer G0.5 with $DACHPt(H_2O)_2$ complex, $G0.5COO(DACHPt)_4$.

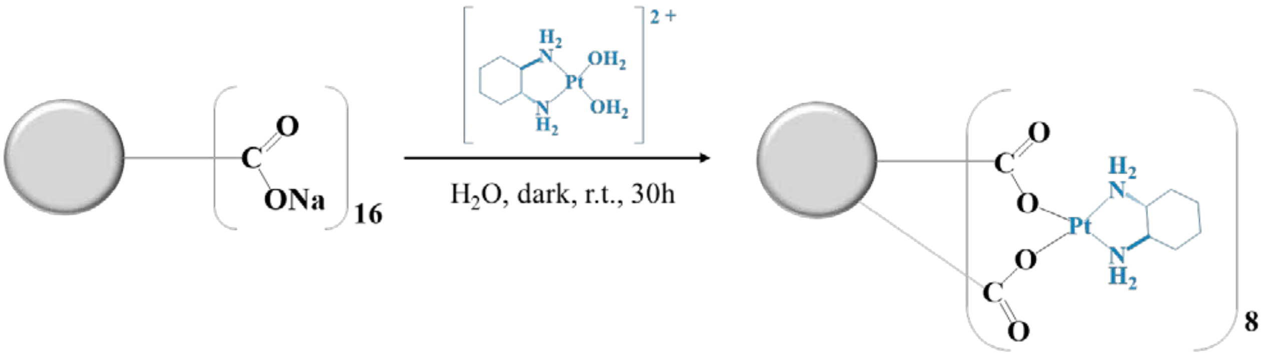

Scheme S4: Representation of the synthesis of anionic PAMAM dendrimer G1.5 with $DACHPt(H_2O)_2$ complex, $G1.5COO(DACHPt)_8$.

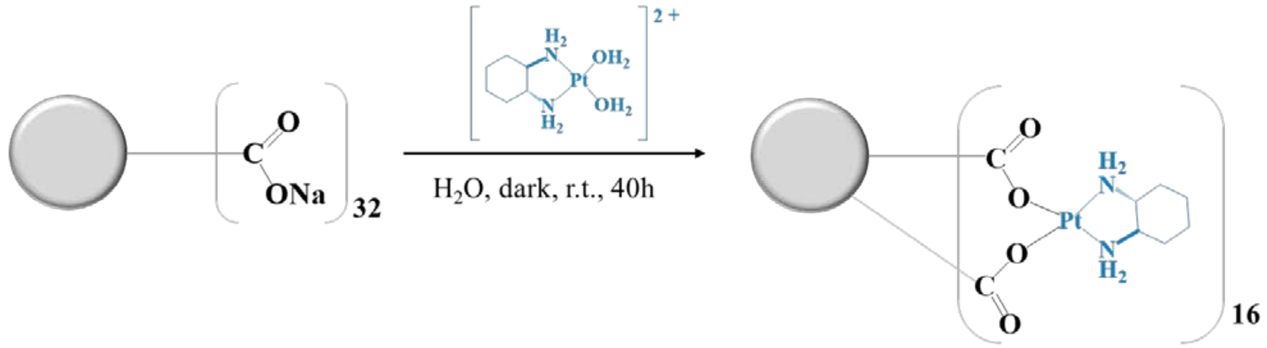

Scheme S5: Representation of the synthesis of anionic PAMAM dendrimer G2.5 with DACHPt(H_2O)$_2$ complex, G2.5COO(DACHPt)$_{16}$.

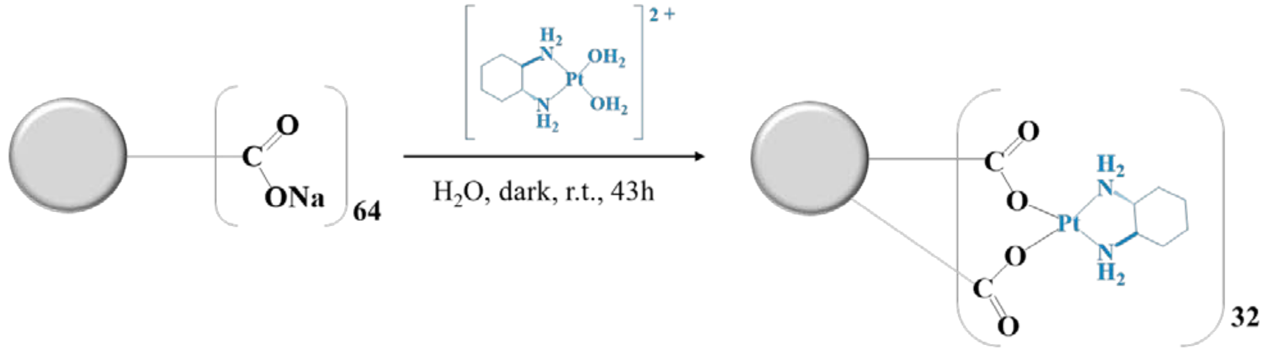

Scheme S6: Representation of the synthesis of anionic PAMAM dendrimer G2.5 with DACHPt(H_2O)$_2$ complex, G3.5COO(DACHPt)$_{32}$.

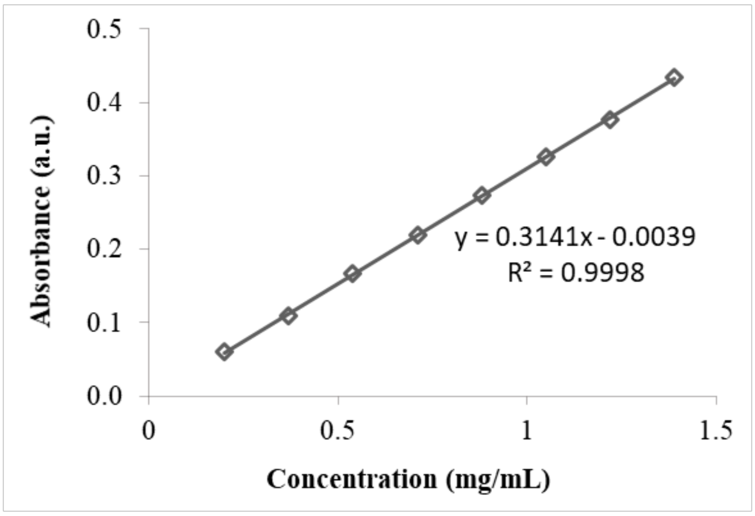

Figure S34: Standard curve of Hg using several dilutions: 0.2; 0.37; 0.54; 0.71; 0.88; 1.05; 1.22 and 1.39mg/mL. The absorbance was measured at 550nm.

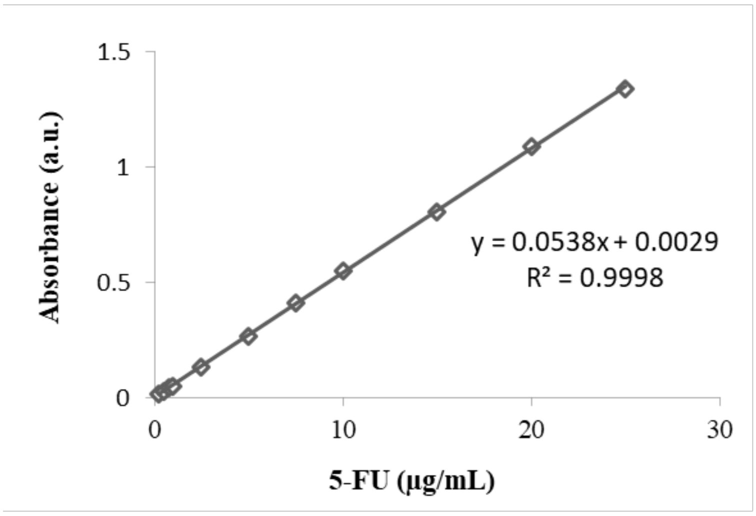

Figure S35: Standard curve of 5-Fluorouracil in ultrapure water using different concentrations: 0.25, 0.5, 0.75, 1, 2.5, 5, 7.5, 10, 15, 20 and 25 μg/mL. The absorbance was measured at 266nm.

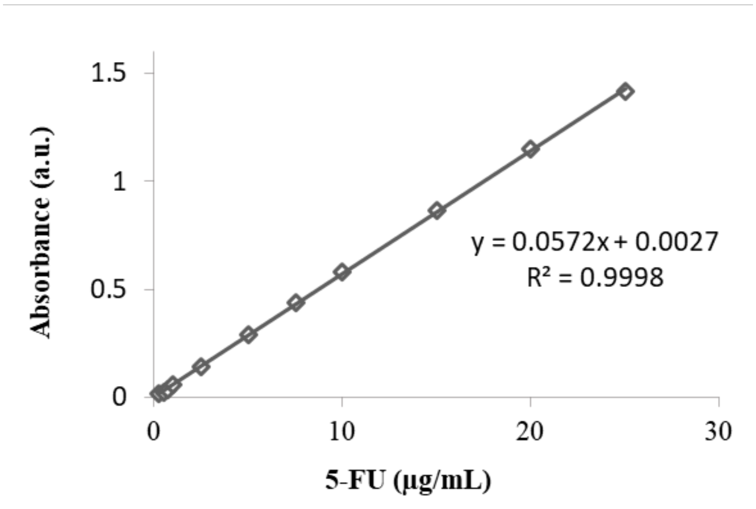

Figure S36: Standard curve of 5-Fluorouracil in PBS 5 using different concentrations: 0.25, 0.5, 0.75, 1, 2.5, 5, 7.5, 10, 15, 20 and 25 μg/mL. The absorbance was measured at 266nm.

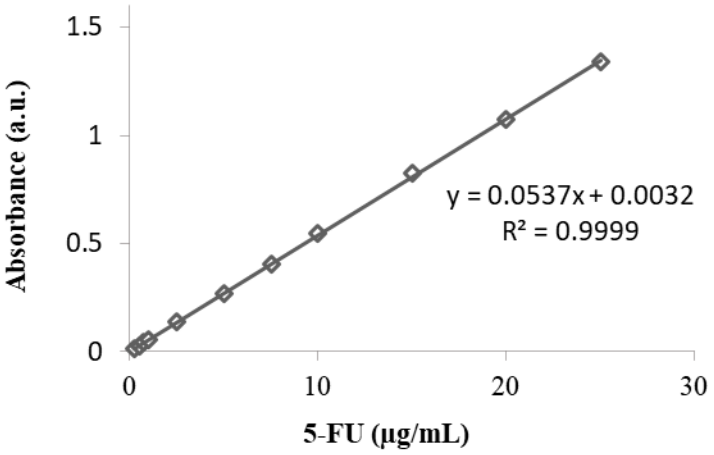

Figure S37: Standard curve of 5-Fluorouracil in PBS 7.4 using different concentrations: 0.25, 0.5, 0.75, 1, 2.5, 5, 7.5, 10, 15, 20 and 25 μg/mL. The absorbance was measured at 266nm.

References

[1] J.R.L. Priqueler, I.S. Butler, F.D. Rochon, An overview of ^{195}Pt nuclear magnetic resonance spectroscopy, Appl. Spectrosc. Rev. 41 (2006) 185–226. https://doi.org/10.1080/05704920600620311.
